# Contemporary Changes in Global Trends in Early-Onset Cancer: Incidence and Mortality (2000–2021)

**DOI:** 10.3390/cancers17172766

**Published:** 2025-08-25

**Authors:** Pojsakorn Danpanichkul, Yanfang Pang, Supapitch Sirimangklanurak, Thanida Auttapracha, Thanawin Pramotedham, Chun Wei Pan, Benjamin Koh, Zhen Yu Wong, Sakditad Saowapa, Shyna Zhuoying Gunalan, Kwanjit Duangsonk, Chanakarn Kanitthamniyom, Donghee Kim, Karn Wijarnpreecha, Amit G. Singal, Daniel Q. Huang, Ju Dong Yang

**Affiliations:** 1 Department of Internal Medicine, Texas Tech University Health Sciences Center, Lubbock, TX 79430, USA; pdanpani@ttuhsc.edu (P.D.); sakditad.saowapa@ttuhsc.edu (S.S.); zen.kanitthamniyom@ttuhsc.edu (C.K.); 2 Affiliated Hospital of Youjiang Medical University for Nationalities, Baise 533000, China; pangyanfang93@163.com; 3 National Immunological Laboratory of Traditional Chinese Medicine, Baise 533000, China; 4 Center for Medical Laboratory Science, Affiliated Hospital of Youjiang Medical University for Nationalities, Baise 533000, China; 5 Faculty of Medicine, Chiang Mai University, Chiang Mai 50200, Thailand; supapitch_siri@cmu.ac.th (S.S.); thanida_a@cmu.ac.th (T.A.); 6 Division of Gastroenterology and Hepatology, Department of Medicine, National University Hospital, Singapore 119228, Singapore; a0133417@u.nus.edu; 7 Department of Internal Medicine, Cook County Health, Chicago, IL 60612, USA; chunwei.pan@cookcountyhealth.org; 8 Yong Loo Lin School of Medicine, National University of Singapore, Singapore 117597, Singapore; e0950819@u.nus.edu (B.K.); e1177562@u.nus.edu (S.Z.G.); 9 Queen’s Medical Centre, Nottingham NG7 2UH, UK; zhen.wong@wales.nhs.uk; 10 Department of Microbiology, Faculty of Medicine, Chiang Mai University, Chiang Mai 50200, Thailand; kwanjit.d@cmu.ac.th; 11 Division of Gastroenterology and Hepatology, Stanford University School of Medicine, Stanford, CA 94305, USA; dhkimmd@stanford.edu; 12 Division of Gastroenterology and Hepatology, Department of Medicine, University of Arizona College of Medicine, Phoenix, AZ 85004, USA; karn.wijarnpreecha2@bannerhealth.com; 13 Division of Gastroenterology and Hepatology, Department of Internal Medicine, Banner University Medical Center, Phoenix, AZ 85006, USA; 14 BIO5 Institute, University of Arizona College of Medicine-Phoenix, Phoenix, AZ 85004, USA; 15 Division of Digestive and Liver Diseases, University of Texas Southwestern Medical Center, Dallas, TX 75390, USA; amit.singal@utsouthwestern.edu; 16 Karsh Division of Gastroenterology and Hepatology, Comprehensive Transplant Center, and Samuel Oschin Comprehensive Cancer Institute, Cedars-Sinai Medical Center, Los Angeles, CA 90048, USA

**Keywords:** cancer, epidemiology, public health, oncology, incidence trends, global burden

## Abstract

**Background/Objectives:** The burden of early-onset cancer (cancer incidence <50 years) has increased globally. Early-onset cancer carries significant societal and economic consequences. We aim to provide updated estimates for incidence and mortality of early-onset cancer. **Methods:** We analyzed the Global Burden of Disease Study 2021 to describe the incidence, death, age-standardized incidence rate (ASIR), age-standardized death rate (ASDR) from early-onset cancer (i.e., cancer in people aged 15–49), and its changes from 2000 to 2021 (reflected as annual percent change [APC]), using Joinpoint regression analysis. **Results:** In 2021, there were 3.16 million new cases and 989,650 deaths from early-onset cancer. From 2000 to 2021, the ASIR of early-onset cancer increased (APC: 0.40%, 95% CI 0.32 to 0.47%), with the highest increase observed in the Eastern Mediterranean region (APC: 1.63%, 95% CI 1.53 to 1.72%), whereas the ASDR decreased across most regions. The ASIR for early-onset cancer had a higher increase in females (APC: 0.62%, 95% CI 0.51 to 0.73%) than males (APC: 0.14%, 95% CI 0.04 to 0.23%). Breast cancer (*n* = 567,900) constituted the highest burden of incident cases, followed by non-melanoma skin (*n* = 507,810) and cervical cancers (n = 307,430). The highest increase in the ASIR was observed in non-melanoma skin cancer (APC:2.18%, 95% CI 1.85–2.51%), thyroid (APC: 1.70%, 95% CI 1.60 to 1.79%), and testicular (APC:1.37%, 95% CI 0.96 to 1.78%) cancers. The greatest increases in the ASDR were observed in peripheral nervous system cancer (APC: 0.97%, 95% CI 0.82 to 1.11%) and multiple myeloma (APC: 0.62%, 95% CI 0.51 to 0.72%). **Conclusions:** From 2000 to 2021, the age-adjusted incidence of early-onset cancer increased, with variation across regions and cancer types. Immediate measures are required at a global, regional, and national level to mitigate the burden of early-onset cancer.

## Simple Summary

Cancer has historically been considered a disease primarily affecting older adults, but the incidence of early-onset cancer, defined as cancer incidence in individuals under 50 years, has been increasing. Cancer diagnosis in young adults has significant medical and financial implications on an individual; furthermore, cancer in younger adults may be diagnosed at later stages than in older adults and have differing tumor biology. Our study explores the change in incidence and death rates across the spectrum of early-onset cancer over the past two decades and elucidates trends that may influence screening.

## 1. Introduction

Historically, cancer has been viewed as a disease primarily affecting older adults, but recent data indicate rising cancer incidence among individuals under 50, termed early-onset cancer [1,2,3,4]. This rise is multifactorial; the growing prevalence of obesity, smoking, and increased alcohol consumption have been identified as contributory factors [5,6,7,8]. In utero exposure to maternal obesity, maternal diabetes, certain medications, or pesticides is hypothesized to be contributory [9,10,11]. Maternal metabolic dysregulation, including obesity and diabetes, may promote a pro-inflammatory intrauterine environment and expose the fetus to elevated growth factors [12]. Additionally, exposure to endocrine-disrupting chemicals found in pesticides can trigger lasting epigenetic changes and alterations in gut microbiota composition [13]. This milieu of factors reshapes metabolic and immune pathways, heightening an individual’s lifelong risk of cancer, including early-onset cancer. Recent data from the United States indicate that early-onset cancer is rising in the United States, with varying trends across cancer types [14,15]. For instance, colorectal cancer incidence has been increasing by 1–2% annually among adults younger than 55 years since the mid-1990s. Breast cancer incidence has also risen more rapidly in women under 50 years (1.1% annually) compared to older women (0.5% annually) [14].

Cancer diagnosis in young to mid-adulthood may lead to psychological stress, isolation, mental disorders, reduced productivity due to interruptions in work, and resultant financial difficulties [16,17,18]. Care for early-onset cancer is multidisciplinary and complex. Patients may be diagnosed at a later cancer stage owing to lower clinical suspicion or being excluded by definition from conventional screening programs. Early-onset cancer is also biologically distinct from cancer in older counterparts. For instance, it has been well described that early-onset colorectal cancer tends to differ histopathologically and in location, often demonstrating more aggressive pathological characteristics [19].

Younger adults are more likely than older cancer patients to experience delays in diagnosis for certain cancers due to the absence of cost-effective early detection methods and the rarity of cancer in this age group [20]. Although cancer in young adults has a lower burden than in older adults, younger adults experience more disability-adjusted life years [21].

Previous work assessing the global burden of early-onset cancer has focused on specific types or geographic regions [22,23]. The Global Burden of Disease Study (GBD) provides comprehensive insights into international trends by systematically estimating multiple diseases across 204 countries and territories, allowing for detailed stratification by disease, sex, geography, and socioeconomic development [24]. Our study aims to analyze the global, regional, and sociodemographic differences in the burden of early-onset cancer and the changes in the past two decades.

## 2. Materials and Methods

### 2.1. Data Source

We utilized data from the GBD 2021 database to measure the impact of 369 diseases and 87 risk factors across 204 countries and territories. We extracted annual incidence, mortality, and age-standardized incidence and death rates for early-onset cancer from 2000 to 2021. This information was accessed on 22 October 2024 via the Global Health Data Exchange (https://ghdx.healthdata.org), a continuously updated online database managed by the Institute for Health Metrics and Evaluation [24]. This study used publicly available, de-identified data and therefore did not require institutional review board approval, in accordance with relevant ethical guidelines and reporting standards.

### 2.2. Definitions and Measures

The general estimation methods for GBD 2021 and specific approaches for estimating cancer have been detailed in prior studies [21]. The data for this study were sourced from population-based cancer registries, vital registration systems, and verbal autopsy studies (verbal autopsy is the preferred approach for routinely determining causes of death in low- and middle-income countries, where civil registration and vital statistics systems are weak or absent, and where medical certification of death causes is limited or lacking) [25,26]. We defined early-onset cancer as cancer diagnoses occurring between the ages of 15 and 49. The upper limit age of 49 is in alignment with the Early-Onset Cancer Initiative definition by the National Cancer Institute [27]. The GBD study does not provide burden estimates for ages from birth to 49 years; we used the closest available age category from the database (15 to 49 years) for our analysis.

For countries lacking cancer mortality data, incidence rates were used to estimate mortality through a modeled mortality-to-incidence ratio (MIR). These MIRs for various cancers were modeled using spatiotemporal Gaussian process regression (ST-GPR). The GBD 2021 study utilized the 10th revision of the International Statistical Classification of Diseases (ICD-10) to classify cancers, with specific ICD-10 codes established in previous research and Supplementary Table S1 [21,28]. Our analysis included the following cancers: lip and oral cavity cancers, nasopharyngeal cancer, other pharyngeal cancer, esophageal cancer, gastric cancer, colorectal cancer, liver cancer, biliary tract cancer, pancreatic cancer, laryngeal cancer, lung cancer (including trachea and bronchus), malignant skin melanoma, non-melanoma skin cancer, soft tissue cancer, bone cancer, breast cancer, cervical cancer, uterine cancer, ovarian cancer, prostate cancer, testicular cancer, kidney cancer, bladder cancer, central nervous system (CNS) cancer, peripheral nervous system (PNS) cancer, eye cancer, thyroid cancer, mesothelioma, Hodgkin lymphoma, non-Hodgkin lymphoma, multiple myeloma, leukemia, and other cancers. Countries were classified into six geographical regions, in alignment with the World Health Organization’s classification of regions: Africa, the Eastern Mediterranean, Europe, the Americas, Southeast Asia, and the Western Pacific [29,30]. Moreover, the regions were categorized by the sociodemographic index (SDI): low, low–middle, middle, high–middle, and high SDIs, with higher SDIs indicating greater socioeconomic development. The SDI in the GBD framework is derived as the geometric mean of three normalized indicators: (1) lag-distributed income per capita, (2) mean educational attainment among individuals aged 15 years and older, and (3) the total fertility rate among women younger than 25 years. Each component is scaled between 0 and 1 to reflect relative development levels across populations. Supplementary Material S1 provides the list of countries categorized by SDI. The data quality by countries is listed in the prior GBD capstone study [31].

Various statistical methods were employed to enhance data reliability, including correcting for misclassification, redistributing nonspecific cause codes, and using algorithms to reduce noise and variation. The Cause of Death Ensemble model (CODEm) assessed age-standardized death rate (ASDR) by age, sex, location, and year, using Bayesian geospatial regression to account for spatial relationships in the data. The expanded methodology can be found in the GBD capstone publications [31,32] and Supplementary Material S2.

### 2.3. Data and Statistical Analysis

The estimated death counts in the GBD 2021 study were reported with 95% uncertainty intervals (UIs), representing the range between the 2.5th and 97.5th ranked values from 1000 draws of the posterior distribution. The GBD methodology systematically propagates uncertainty, derived not only from sampling variability but also from model specification, covariate selection, and parameter estimation, by generating a distribution of posterior draws for each quantity. An uncertainty interval represents the range within which an estimate is likely to fall, indicating the level of confidence in that estimate. In the GBD study, each estimate is generated using random sampling from distributions rather than relying on single-point values for inputs, data adjustments, and model selection. Wider uncertainty intervals often arise from limited or inconsistent data, and small sample sizes, whereas narrower intervals typically reflect abundant, high-quality, and consistent data. Age-standardized rates (ASRs) were calculated using the direct method applied to GBD 2021 population estimates.

To assess changes in ASRs from 2000 to 2021, the annual percent change (APC) and its 95% confidence interval (CI) were calculated. Statistical analysis was conducted using the Joinpoint regression program (version 4.9.1.0) from the National Cancer Institute. An increasing trend is identified if both the APC and the lower bound of its 95% CI are positive, while a declining trend is indicated when both the APC and the upper bound of its 95% CI are negative. Given the impact of the COVID-19 pandemic on disease burden estimates, we also compared analyses for the periods 2000–2021 and 2000–2019 [33,34,35].

## 3. Results

### 3.1. Global Burden of Early-Onset Cancer

**Figure 1:**
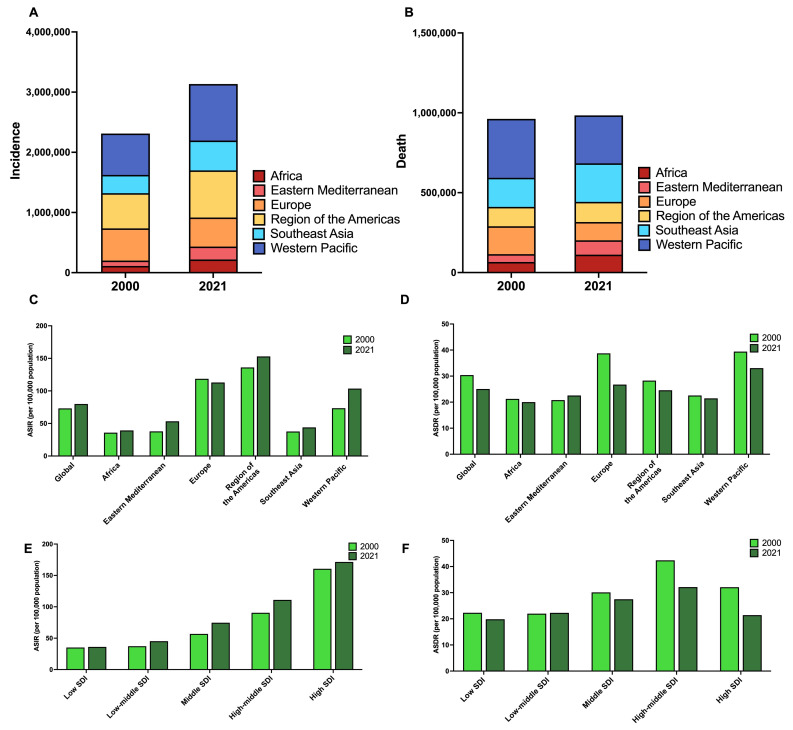
(**A**) Number of incident cancer cases in patients aged 15–49 in 2000 and 2021, stratified by the World Health Organization region. (**B**) Number of cancer deaths among patients aged 15–49 in 2000 and 2021, stratified by the World Health Organization region. (**C**) Age-standardized incidence rates (per 100,000 population) of cancer among patients aged 15–49 in 2000 and 2021, stratified by the World Health Organization region. (**D**) Age-standardized death rates (per 100,000 population) of cancer among patients aged 15–49 in 2000 and 2021, stratified by the World Health Organization region. (**E**) Age-standardized incidence rates (per 100,000 population) of cancer among patients aged 15–49 in 2000 and 2021, stratified by sociodemographic index. (**F**) Age-standardized death rates (per 100,000 population) of cancer among patients aged 15–49 in 2000 and 2021, stratified by sociodemographic index. Legend: ASDR, age-standardized death rate; ASIR, age-standardized incidence rate; SDI, sociodemographic index.

Globally, in 2021, the number of incidents of early-onset cancer cases and deaths were 3.16 million (95% UI: 2.98 million to 3.34 million) and 989,650 (95% UI: 927,780 to 1.05 million), respectively (Table 1 and Figure 1A,B). In 2021, the estimated early-onset cancer ASIR and ASDR were 79.91 (95% UI: 75.52 to 84.60) per 100,000 population and 25.06 (95% UI: 23.50 to 26.61) per 100,000 population, respectively (Table 1 and Figure 1C,D). Between 2000 and 2021, the number of incident cases of early-onset cancer and deaths increased by 35% and 2%, respectively. Over this period, the ASIR (APC: 0.40%, 95% CI: 0.32 to 0.47%) from early-onset cancer increased, but the ASDR (APC: −0.91%, 95% CI: −1.02 to −0.80%) decreased (Table 1). Supplementary Table S2 presents early-onset cancer data for 2019–2021, along with trend comparisons for the periods 2000–2019 and 2000–2021.

The burden of early-onset cancer, stratified by age group, is listed in Supplementary Table S3. In 2021, the ASIR increased with age, from 11.4 per 100,000 population in the 15–19 age group to 238.3 per 100,000 population in those aged 45–49. The increases in the ASIR across certain 5-year age groups were significant, particularly among individuals aged 20–24 (APC 0.28%, 95% CI: 0.18 to 0.37%), 25–29 (APC 0.53%, 95% CI: 0.24 to 0.81%), and 30–34 (APC: 0.50%, 95% CI: 0.11 to 0.90%), while non-significant changes were observed between older 5-year age groups within ages 35–49. ASDR decreased significantly across all age groups over the study period, with the steepest annual decline observed in the 45–49 age group (APC −1.48%, 95% CI: −1.68 to −1.29). Essentially, decreasing mortality was noted in spite of the rising incidence of early-onset cancer (Supplementary Table S3).

### 3.2. The Burden of Early-Onset Cancer, by Sex

In 2021, there were 1.90 and 1.25 million early-onset cancer cases in females and males, respectively (Table 1). There were 508,560 and 481,080 early-onset cancer deaths in females and males, respectively. The ASIR in females and males was 97.73 (95% UI: 91.18 to 104.52) and 62.54 (95% UI: 58.16 to 68.02) per 100,000 population, respectively. The ASDR in females was 26.10 (95% UI: 24.15 to 28.12), while the ASDR in males was 24.06 (95% UI: 21.95 to 26.39) per 100,000 population (Table 1). The ASIR increased to a greater degree in females (APC: 0.62%, 95% CI: 0.51 to 0.73%) compared to males (APC: 0.14%, 95% CI: 0.04 to 0.23%) from 2000 to 2021, while the ASDR decreased in both sexes, with a greater decline among males (APC: −1.23%, 95% CI: −1.36 to −1.09%) than females (APC: −0.61%, 95% CI: −0.76 to −0.46%) (Table 1). The burden of disease between sex stratified by region and SDI is listed in Supplementary Tables S4 and S5. Across most regions and SDI levels, it is striking that early-onset cancer ASIR and ASDR were higher in females, except in the Western Pacific region and in middle- to high–middle SDI countries, where males exhibited a higher ASDR (while still demonstrating a lower ASIR).

### 3.3. The Burden of Early-Onset Cancer, by the World Health Organization Region

In 2021, the highest frequency of early-onset cancer cases (*n* = 939,520) and deaths (*n* = 300,000) were observed in the Western Pacific region (Table 1 and Figure 1A,B). The highest ASIR was observed in the Americas, with a value of 152.97 (95% UI: 143.22 to 162.91) per 100,000 population, whereas the highest ASDR was observed in the Western Pacific, with a value of 33.07 (95% UI: 28.24 to 38.50) per 100,000 population (Table 1 and Figure 1C,D). From 2000 to 2021, the ASIR of early-onset cancer increased in most WHO regions, with the highest increase observed in the Eastern Mediterranean region (APC: 1.63%, 95% CI: 1.53 to 1.72%). In contrast, the ASDR of early-onset cancer decreased in most WHO regions, with increases observed only in the Eastern Mediterranean region (APC: 0.40%, 95% CI: 0.32 to 0.48%) (Table 1).

### 3.4. The Burden of Early-Onset Cancer, by Sociodemographic Index

In 2021, middle-SDI countries had the highest number of early-onset cancer cases (*n* = 937,400) and deaths (*n* = 344,910) (Table 1). High-SDI countries had the highest ASIR of early-onset cancer (171.54; 95% UI: 162.25 to 181.82), while high–middle SDI countries had the highest ASDR (32.14; 95% UI: 28.99 to 35.71) per 100,000 population (Table 1 and Figure 1E,F). Between 2000 and 2021, early-onset cancer ASIRs increased in most countries, with the highest rise observed in middle-SDI countries (APC: 1.23%, 95% CI: 0.99 to 1.46%). In the same timeframe, the ASDR decreased in most SDI strata, except low-middle SDI countries, which had a stable ASDR (Table 1).

### 3.5. The Burden of Early-Onset Cancer, by Cancer Type

**Figure 2:**
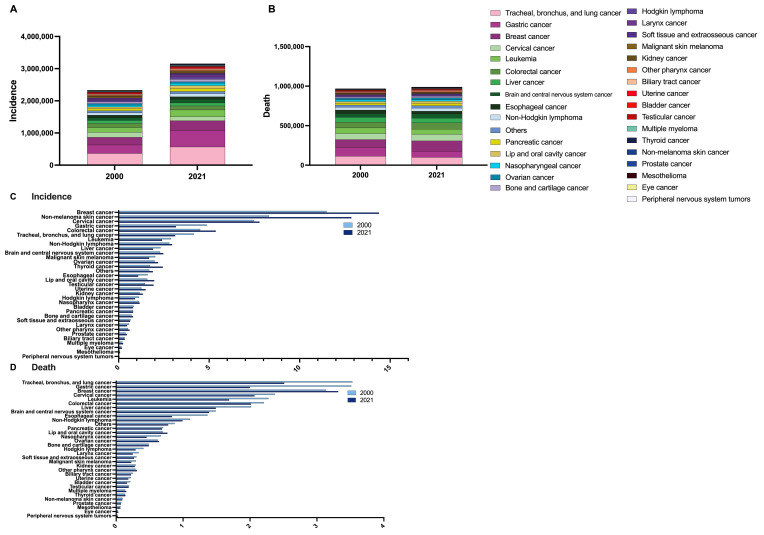
(**A**) Number of incident cancer cases among patients aged 15–49 in 2000 and 2021, stratified by cancer type. (**B**) Number of cancer deaths in patients aged 15–49 in 2000 and 2021, stratified by cancer type. (**C**) Age-standardized incidence rates (per 100,000 population) of cancer among patients aged 15–49 in 2000 and 2021, stratified by cancer type. (**D**) Age-standardized death rates (per 100,000 population) of cancer among patients aged 15–49 in 2000 and 2021, stratified by cancer type.

**Figure 3:**
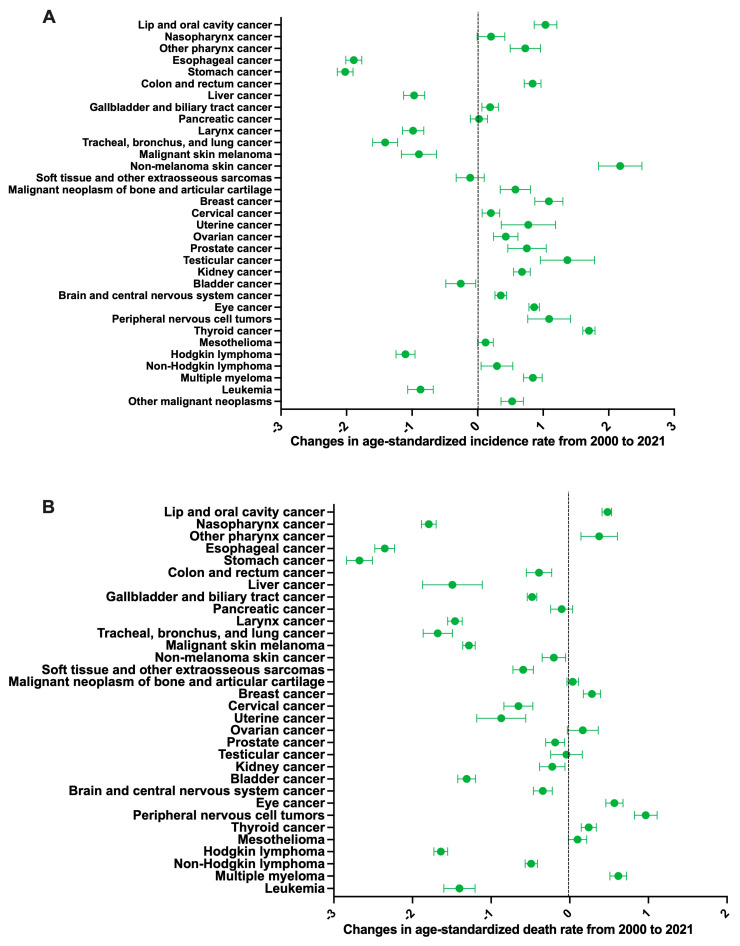
(**A**) Annual percent change in age-standardized incidence rates (per 100,000 population) of cancer in patients aged 15–49 from 2000 to 2021, stratified by type of cancer. (**B**) Annual percent change in age-standardized death rates (per 100,000 population) of cancer in patients aged 15–49 from 2000 to 2021, stratified by type of cancer.

Breast cancer accounted for the highest number of incident early-onset cancer cases (*n* = 567,900) and deaths (*n* = 131,020) (Table 2 and Figure 2A,B). The other cancers with high incidence include non-melanoma skin cancer (507,810), cervical cancer (307,430), colorectal cancer (211,890), and gastric cancer (125,120) (Table 2 and Figure 2A). Other cases with high mortality include lung (including tracheal and bronchial) cancer (99,130), cervical cancer (81,640), colorectal cancer (79,500), and gastric cancer (78,870) (Table 2 and Figure 2B). The ASIR and ASDR are shown in Figure 2C,D. Between 2000 and 2021, the ASIR of early-onset cancer increased in lip and oral cavity cancers (APC: 1.04%, 95% CI: 0.86 to 1.21%), pharyngeal cancer (APC: 0.73%, 95% CI: 0.50 to 0.96%), colorectal cancer (APC: 0.84%, 95% CI: 0.71 to 0.97%), liver cancer from metabolic dysfunction-associated steatohepatitis (APC: 0.26%, 95% CI: 0.16 to 0.35%), biliary tract cancer (APC: 0.19%, 95% CI: 0.06 to 0.32%), non-melanoma skin cancer (APC: 2.18%, 95% CI: 1.85 to 2.51%), bone cancer (APC: 0.58%, 95% CI: 0.34 to 0.81%), breast cancer (APC: 1.09%, 95% CI: 0.87 to 1.30%), cervical cancer (APC: 0.20%, 95% CI: 0.07 to 0.34%), uterine cancer (APC: 0.77%, 95% CI: 0.36 to 1.19%), ovarian cancer (APC: 0.43%, 95% CI: 0.24 to 0.62%), prostate cancer (APC: 0.75%, 95% CI: 0.46 to 1.05%), testicular cancer (APC: 1.37%, 95% CI: 0.96 to 1.78%), kidney cancer (APC: 0.68%, 95% CI: 0.55 to 0.80%), CNS (APC: 0.35%, 95% CI: 0.26 to 0.44%), eye cancer (APC: 0.86%, 95% CI: 0.78 to 0.94%), PNS (APC: 1.09%, 95% CI: 0.76 to 1.42%), thyroid cancer (APC: 1.70%, 95% CI: 1.60 to 1.79%), mesothelioma (APC: 0.12%, 0.00 to 0.24%) multiple myeloma (APC: 0.84%, 95% CI: 0.70 to 0.99%), non-Hodgkin lymphoma (APC: 0.29%, 95% CI: 0.05 to 0.54%), and other cancers (APC: 0.53%, 95% CI: 0.36 to 0.70%) (Table 2 and Figure 3A).

The ASDR increased for lip and oral cavity cancers (APC: 0.47%, 95% CI: 0.42 to 0.51%), pharyngeal cancer (APC: 0.37%, 95% CI: 0.14 to 0.61%), breast cancer (APC: 0.28%, 95% CI: 0.17 to 0.39%), eye cancer (APC: 0.57%, 95% CI: 0.46 to 0.68%), PNS (APC: 0.97%, 95% CI: 0.82 to 1.11%), thyroid cancer (APC: 0.24%, 95% CI: 0.15 to 0.34%), and multiple myeloma (APC: 0.62%, 95% CI: 0.51 to 0.72%) (Table 2 and Figure 3B).

### 3.6. The Burden of Early-Onset Cancer, by Country

**Figure 4:**
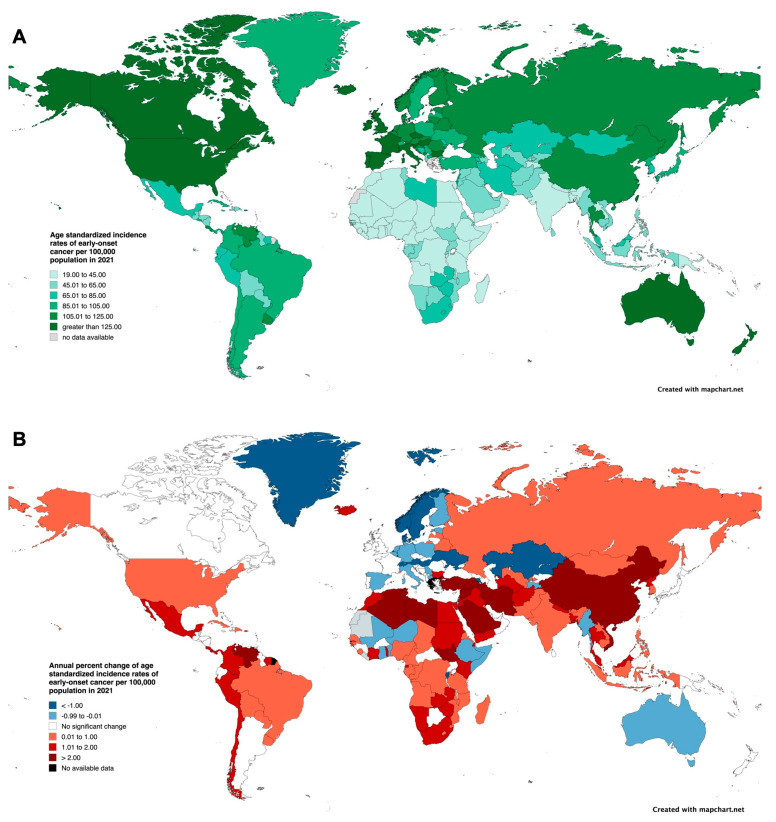
(**A**) Age-standardized incidence rates (per 100,000 population) of cancer among patients aged 15–49 in 2021, stratified by country/territory. (**B**) Annual percent change in age-standardized incidence rates (per 100,000 population) of cancer among patients aged 15–49 from 2000 to 2021, stratified by country/territory.

The ASIR ranged from 19.65 (95% UI: 13.77 to 27.31) incident cases per 100,000 population in Niger to 310.89 (95% UI: 284.72 to 339.96) incident cases per 100,000 population in the United States (Figure 4A and Supplementary Table S6). The highest increase was observed in Saudi Arabia (APC: 3.61%, 95% CI: 3.51 to 3.71%), Iran (APC: 3.11%, 95% CI: 2.90 to 3.32%), and Libya (2.83%, 95% CI: 2.45 to 3.20%) (Figure 4B and Supplementary Table S6).

## 4. Discussion

### 4.1. Main Findings

In 2021, our study identified 3.16 million new early-onset cancer cases and 989,650 related deaths worldwide. Compared to the previous GBD 2019 study [36], which reported a 79% increase in early-onset cancer incidence and a 28% rise in deaths from 1990 to 2019, our analysis from 2000 to 2021 shows more modest increases, namely 35% in incidence and 2% in mortality. This may indicate a leveling off in death rates, potentially reflecting improvements in detection and treatment. The differences could also stem from variations in methodology (e.g., updated GBD approaches), study periods, or data quality [31]. It is also plausible that data quality would have been affected during the COVID-19 pandemic due to under-reporting. Breast cancer has emerged as the leading cause of mortality and the most common cancer by incidence among early-onset cancers. Although mortality rates have generally declined for most cancers in this age group, breast cancer has continued to demonstrate increasing incidence and death rates over the past two decades. Over this same period, thyroid cancer, non-melanoma skin cancer, and testicular cancer exhibited the greatest increases in incidence among early-onset cancers. Regionally, the ASIR of cancer in early-onset cancer increased in most of the geographic regions, whereas the ASDR decreased in all regions except the Eastern Mediterranean.

### 4.2. Findings in the Context of Current Literature

These findings build upon prior research that analyzed early-onset cancer trends in the GBD study up to 2019 [37]. They are also consistent with the recent Surveillance, Epidemiology, and End Results (SEER) and GLOBOCAN studies, which reported increases in the ASIRs of early-onset cancers [1,38]. This study further enriches these findings by providing updated global, regional, and national patterns through 2021, classified by sex, region, and country development index.

While mortality rates in most early-onset cancers have declined, breast cancer stands out, with rising incidence and death rates over the past two decades. Apparent increases in incidence may be partially attributed to detection bias from improved screening, but multiple other factors are postulated to have led to actual increases. Among these are metabolic risk factors [39,40], prior cancer treatments, including chest wall radiation and alkylating agents that may have led to secondary breast cancer [41,42], physical inactivity, westernized diets, and earlier age at menarche.

Moving beyond breast cancer, non-melanoma skin cancers, thyroid cancer, testicular tumors, and peripheral nervous system cell tumors are also notable for increases in the ASIR. While hereditary cancer syndromes (such as Lynch syndrome, BRCA-related breast and ovarian cancer) are established contributors to early-onset cancer risk, they account for only a minority of cases [43,44]. Sociodemographic changes, including improved healthcare access and heightened medical literacy, may partly explain the increase [45,46]. Hormonal influences such as earlier puberty onset or exogenous hormone exposure, including supplements, likely play a role in testicular cancer [47,48,49]. Intriguingly, the ASIR of non-melanoma skin cancer is increasing despite improvements in awareness of sun-protective behavior; hence, this could be more likely due to increased detection [50]. Similarly, increased thyroid cancer incidence is primarily due to the detection of incidental tumors through the widespread use of modern medical imaging; however, the secondary role of environmental exposures and lifestyle changes, such as radiation exposure and obesity, cannot be discounted [51,52,53]. The reasons underlying the rise in peripheral nerve tumors remain unclear. While specific drivers are expected to differ between cancer types, common contributing factors likely include improved detection, evolving environmental and lifestyle exposures, genetic predispositions, and the interplay between these factors.

On the other hand, gastric, esophageal, and tracheal/bronchus/lung cancers have shown the greatest declines in incidence over the past two decades. The positive association between tobacco exposure and the incidence of these cancers is well known. Correspondingly, the success of public health policies aimed at reducing smoking has likely played a major role in this downward trend [54,55,56]. Advancements in managing gastroesophageal reflux disease and enhanced endoscopic surveillance have likely reduced the progression of precursor pre-malignant lesions into esophageal and gastric cancer [57,58]. Stricter regulations on industrial pollutants have also played a role in lowering lung cancer risk [59].

Regionally, the highest increase in incidence and death rates is observed in the Eastern Mediterranean. It is postulated that rising alcohol use and metabolic risk factors may contribute [8,60].

### 4.3. Implications for Clinical Practice and Future Research

This study provides updated global, regional, and national evidence that the incidence of early-onset cancers is increasing and highlights several disparities. The increase in early-onset cancer disproportionately occurred in cancers of the lip and oral cavity, nasopharynx, colorectum, biliary tract, female reproductive tract, male reproductive tract, thyroid, as well as in non-Hodgkin lymphoma and non-melanoma skin cancers. Females experienced a disproportionately increased incidence rate of early-onset cancer, predominantly attributable to breast cancer [61]. Separately, SEER data have demonstrated that early-onset cancer incidence approaches near parity with men after excluding breast cancer, highlighting its dominant influence [61]. Beyond the aforementioned factors, which are postulated to contribute to rising breast cancer incidence, our understanding likely remains incomplete. Hormonal factors, the rise in obesity, and the metabolic syndrome may not fully account for the observed increases. For instance, premenopausal breast cancer risk is lower in women with higher childhood BMI [62]. Screening alone also fails to explain these trends: population-wide screening in U.S. women under 50 years during the 2001 to 2019 period was limited to breast and cervical cancer, and the increase in breast cancer incidence was evident even in women aged 25–39, below routine screening thresholds [61]. The significant impact of early-onset breast cancer and the possibility of yet unidentified etiological factors underscore the urgent need for novel etiologic research beyond traditional reproductive or behavioral explanations.

Generally speaking, healthcare professionals and policymakers should be informed about the increasing incidence of early-onset cancer, and investigations for possible malignancy need to be considered even in younger patients. Addressing early-onset cancer necessitates approaches that capture the complex interplay of biological, behavioral, and environmental influences throughout the life course. Systems epidemiology, which models how these factors interact at both individual and population levels, provides a valuable framework to explore underlying drivers and assess the potential impact of prevention strategies, especially in light of evolving exposomes and early-life risk exposures [63,64,65]. By combining effective public health approaches with robust research initiatives, the field can work towards significantly reducing the burden of early-onset cancer and improving outcomes for individuals affected by these diseases.

### 4.4. Limitations

This study has several strengths, including its large-scale data from over 204 countries and territories, updated through 2021, and detailed subgroup analyses by cancer type. However, it also has limitations. This study is constrained by the quality of each country’s registry data, which is the inherent limitation of the GBD study [31,66]. A limitation of this study is the variability in registry completeness and data quality, with modeled estimates used in data-sparse regions. For example, a prior study comparing gastrointestinal cancer data revealed substantial discrepancies between the GBD study and the GLOBOCAN database, particularly in the Southeast Asia region [67]. This may introduce heterogeneity and reduce accuracy, particularly in lower SDI countries and during the COVID-19 pandemic [68]. However, our trend analysis indicates no change in the trend when comparing the periods 2000–2021 and 2019–2021. The GBD 2021 study did not assess the subgroup of some cancers separately, such as tracheal, bronchial, and lung cancers, which are categorized together, and lip and oral cavity cancers. While ages 15 to 49 are commonly used to define early-onset cancers, this broad range includes heterogeneous subgroups with distinct risk profiles and cancer patterns. We were unable to stratify breast cancer cases into pre- and post-menopausal categories due to the lack of menopausal status information in the GBD dataset. Future studies should consider stratified analyses using different age bands or hormonal status to improve epidemiological precision and uncover age-specific trends [69]. Additionally, the GBD 2021 lacks data on cancer histological subgroups, highlighting the need for future research to assess the global burden of these subtypes [31]. Lastly, we were unable to access the pre-processed data used to calculate the mortality-to-incidence ratio.

## 5. Conclusions

The incidence of early-onset cancers has risen over the past two decades. While breast cancer had the highest number of new cases, thyroid cancer, non-melanoma skin cancer, and testicular cancer demonstrated the fastest-growing incidence rates among all early-onset cancers. These findings have important implications for shaping surveillance strategies and prioritizing funding for research and prevention efforts.

## Figures and Tables

**Table 1:** Incidence, death, age-standardized incidence rates, age-standardized death rates from cancer in patients aged 15–49 years by sex, region, and sociodemographic index.

	Incidence				Death			
	2021 Incidence (95% UI)	2021 Age-Standardized Incidence Rate (95% UI) per 100,000 Population	2000 to 2021 Annual Percent Change (95% CI)	*p*	2021 Death (95% UI)	2021 Age-Standardized Death Rate (95% UI) per 100,000 Population	2000 to 2021 Annual Percent Change (95% CI)	*p*
Total	3.16 million (2.98 million to 3.34 million)	79.91 (75.52 to 84.6)	0.4 (0.32 to 0.47)	<0.001	989,650 (927,780 to 1.05 million)	25.06 (23.5 to 26.61)	−0.91 (−1.02 to −0.8)	<0.001
Sex								
Female	1.90 million (1.78 million to 2.04 million)	97.73 (91.18 to 104.52)	0.62 (0.51 to 0.73)	<0.001	508,560 (470,680 to 548,040)	26.1 (24.15 to 28.12)	−0.61 (−0.76 to −0.46)	<0.001
Male	1.25 million (1.16 million to 1.36 million)	62.54 (58.16 to 68.02)	0.14 (0.04 to 0.23)	0.005	481,080 (439,040 to 527,770)	24.06 (21.95 to 26.39)	−1.23 (−1.36 to −1.09)	<0.001
Region								
Africa	219,740 (186,650 to 256,050)	39.27 (33.36 to 45.76)	0.44 (0.33 to 0.55)	<0.001	112,030 (93,830 to 132,280)	20.02 (16.77 to 23.64)	−0.28 (−0.35 to −0.2)	<0.001
Eastern Mediterranean	213,290 (190,960 to 237,530)	53.31 (47.73 to 59.37)	1.63 (1.53 to 1.72)	<0.001	90,230 (79,320 to 102,070)	22.55 (19.82 to 25.51)	0.4 (0.32 to 0.48)	<0.001
Europe	482,880 (462,480 to 501,900)	112.95 (108.18 to 117.4)	−0.28 (−0.58 to 0.02)	0.067	114,380 (109,450 to 119,340)	26.76 (25.6 to 27.92)	−1.7 (−1.81 to −1.58)	<0.001
Region of the Americas	784,530 (734,540 to 835,510)	152.97 (143.22 to 162.91)	0.6 (0.35 to 0.84)	<0.001	125,910 (120,080 to 132,310)	24.55 (23.41 to 25.8)	−0.64 (−0.8 to −0.48)	<0.001
Southeast Asia	495,200 (454,350 to 538,090)	44.01 (40.38 to 47.82)	0.75 (0.6 to 0.9)	<0.001	241,410 (221,710 to 262,210)	21.45 (19.7 to 23.3)	−0.24 (−0.36 to −0.13)	<0.001
Western Pacific	939,520 (825,400 to 1,078,110)	103.57 (90.99 to 118.84)	1.62 (1.38 to 1.86)	<0.001	300,000 (256,190 to 349,280)	33.07 (28.24 to 38.5)	−0.86 (−1 to −0.73)	<0.001
SDI #								
Low SDI	195,800 (167,910 to 223,660)	36.1 (30.96 to 41.24)	0.14 (0.01 to 0.27)	0.037	107,630 (91,850 to 123,550)	19.84 (16.93 to 22.78)	−0.57 (−0.66 to −0.49)	<0.001
Low–middle SDI	459,400 (419,350 to 500,330)	45.21 (41.26 to 49.23)	0.92 (0.74 to 1.1)	<0.001	226,440 (207,070 to 246,470)	22.28 (20.38 to 24.25)	0.06 (−0.11 to 0.23)	0.493
Middle SDI	937,400 (869,360 to 1,013,840)	74.69 (69.27 to 80.78)	1.23 (0.99 to 1.46)	<0.001	344,910 (318,640 to 375,670)	27.48 (25.39 to 29.93)	−0.48 (−0.66 to −0.3)	<0.001
High–middle SDI	698,740 (644,550 to 767,280)	110.99 (102.38 to 121.87)	0.85 (0.71 to 0.98)	<0.001	202,340 (182,490 to 224,840)	32.14 (28.99 to 35.71)	−1.3 (−1.56 to −1.04)	<0.001
High SDI	861,540 (814,880 to 913,140)	171.54 (162.25 to 181.82)	0.36 (0.25 to 0.47)	<0.001	107,480 (104,660 to 110,340)	21.4 (20.84 to 21.97)	−1.94 (−2.1 to −1.78)	<0.001

Abbreviations: CI, confidence interval; SDI, sociodemographic index; UI, uncertainty interval. # The index of countries according to SDI is found in Supplementary Material S1. * The *p*-value indicates the *p*-value of age-standardized rate change from 2000 to 2021; a *p*-value less than 0.05 indicates statistical significance.

**Table 2:** Incidence, deaths, age-standardized incidence rates, age-standardized death rates of cancer in patients aged 15–49 years, by cancer type.

	Incidence				Death			
	2021 Incidence (95% UI)	2021 Age-Standardized Incidence Rate (95% UI) per 100,000 Population	2000 to 2021 Annual Percent Change (95% CI)	*p*	2021 Death (95% UI)	2021 Age-Standardized Death Rate (95% UI) per 100,000 Population	2000 to 2021 Annual Percent Change (95% CI)	*p *
Lip and oral cavity cancers	77,650 (68,360 to 84,640)	1.97 (1.73 to 2.14)	1.04 (0.86 to 1.21)	<0.001	30,270 (26,150 to 33,420)	0.77 (0.66 to 0.85)	0.47 (0.42 to 0.51)	<0.001
Nasopharynx cancer	45,880 (39,880 to 52,770)	1.16 (1.01 to 1.34)	0.2 (−0.01 to 0.41)	0.057	17,970 (15,840 to 20,060)	0.46 (0.4 to 0.51)	−1.79 (−1.89 to −1.7)	<0.001
Other pharynx cancer	23,930 (21,930 to 25,970)	0.61 (0.56 to 0.66)	0.73 (0.5 to 0.96)	<0.001	12,270 (10,930 to 13,580)	0.31 (0.28 to 0.34)	0.37 (0.14 to 0.61)	0.002
Esophageal cancer	42,700 (38,140 to 47,970)	1.08 (0.97 to 1.21)	−1.89 (−2.01 to −1.77)	<0.001	32,920 (29,480 to 36,950)	0.83 (0.75 to 0.94)	−2.35 (−2.48 to −2.23)	<0.001
Gastric cancer	125,120 (107,270 to 144,780)	3.17 (2.72 to 3.67)	−2.02 (−2.14 to −1.9)	<0.001	78,870 (68,700 to 90,840)	2 (1.74 to 2.3)	−2.67 (−2.84 to −2.51)	<0.001
Colorectal cancer	211,890 (193,830 to 231,270)	5.37 (4.91 to 5.86)	0.84 (0.71 to 0.97)	<0.001	79,500 (72,700 to 86,540)	2.01 (1.84 to 2.19)	−0.39 (−0.55 to −0.23)	<0.001
Liver cancer	74,950 (65,250 to 87,630)	1.9 (1.65 to 2.22)	−0.97 (−1.13 to −0.81)	<0.001	58,830 (51,340 to 68,520)	1.49 (1.3 to 1.74)	−1.49 (−1.87 to −1.11)	<0.001
Liver cancer due to alcohol use	8290 (5770 to 11,340)	0.21 (0.15 to 0.29)	−0.1 (−0.18 to −0.03)	0.008	6590 (4570 to 9040)	0.17 (0.12 to 0.23)	−0.38 (−0.49 to −0.27)	<0.001
Liver cancer due to hepatitis B	50,880 (42,110 to 61,740)	1.29 (1.07 to 1.56)	−1.2 (−1.39 to −1.02)	<0.001	39,620 (33,030 to 47,870)	1 (0.84 to 1.21)	−1.78 (−2.11 to −1.44)	<0.001
Liver cancer due to hepatitis C	7130 (5430 to 9300)	0.18 (0.14 to 0.24)	−0.65 (−0.73 to −0.57)	<0.001	5650 (4290 to 7440)	0.14 (0.11 to 0.19)	−0.93 (−1.13 to −0.73)	<0.001
Liver cancer due to MASH	4300 (3310 to 5510)	0.11 (0.08 to 0.14)	0.26 (0.16 to 0.35)	<0.001	3550 (2730 to 4560)	0.09 (0.07 to 0.12)	−0.07 (−0.23 to 0.09)	0.417
Liver cancer due to other causes	4360 (3380 to 5600)	0.11 (0.09 to 0.14)	−1.07 (−1.22 to −0.92)	<0.001	3420 (2650 to 4410)	0.09 (0.07 to 0.11)	−1.5 (−1.8 to −1.2)	<0.001
Biliary tract cancer	13,610 (10,670 to 15,790)	0.34 (0.27 to 0.4)	0.19 (0.06 to 0.32)	0.003	8780 (6940 to 10,240)	0.22 (0.18 to 0.26)	−0.48 (−0.54 to −0.42)	<0.001
Pancreatic cancer	31,530 (28,670 to 34,520)	0.8 (0.73 to 0.87)	0.02 (−0.11 to 0.15)	0.767	27,000 (24,490 to 29,600)	0.68 (0.62 to 0.75)	−0.1 (−0.24 to 0.04)	0.151
Larynx cancer	18,430 (16,900 to 20,210)	0.47 (0.43 to 0.51)	−0.99 (−1.15 to −0.82)	<0.001	9780 (8900 to 10,870)	0.25 (0.23 to 0.28)	−1.46 (−1.55 to −1.37)	<0.001
Tracheal, bronchus, and lung cancer	123,410 (109,790 to 137,020)	3.13 (2.78 to 3.47)	−1.41 (−1.6 to −1.22)	<0.001	99,130 (88,230 to 109,950)	2.51 (2.23 to 2.78)	−1.68 (−1.87 to −1.49)	<0.001
Malignant skin melanoma	66,620 (60,300 to 70,410)	1.69 (1.53 to 1.78)	−0.9 (−1.16 to −0.63)	<0.001	8930 (7240 to 10,220)	0.23 (0.18 to 0.26)	−1.28 (−1.36 to −1.2)	<0.001
Non-melanoma skin cancer	507,810 (419,540 to 601,460)	12.86 (10.62 to 15.23)	2.18 (1.85 to 2.51)	<0.001	3660 (3050 to 4100)	0.09 (0.08 to 0.1)	−0.2 (−0.35 to −0.05)	0.008
Soft tissue and other extraosseous cancer	25,160 (21,500 to 31,360)	0.64 (0.54 to 0.79)	−0.11 (−0.33 to 0.1)	0.3	10,580 (9010 to 13,500)	0.27 (0.23 to 0.34)	−0.59 (−0.72 to −0.46)	<0.001
Bone and articular cartilage cancer	31,390 (26,020 to 35,620)	0.79 (0.66 to 0.9)	0.58 (0.34 to 0.81)	<0.001	19,260 (16,200 to 22,420)	0.49 (0.41 to 0.57)	0.04 (−0.04 to 0.11)	0.303
Breast cancer	567,900 (530,270 to 610,270)	14.38 (13.43 to 15.46)	1.09 (0.87 to 1.3)	<0.001	131,020 (121,840 to 140,900)	3.32 (3.09 to 3.57)	0.28 (0.17 to 0.39)	<0.001
Cervical cancer	307,430 (280,670 to 335,690)	7.79 (7.11 to 8.5)	0.2 (0.07 to 0.34)	0.003	81,640 (73,780 to 90,480)	2.07 (1.87 to 2.29)	−0.65 (−0.84 to −0.47)	<0.001
Uterine cancer	58,860 (50,770 to 65,450)	1.49 (1.29 to 1.66)	0.77 (0.36 to 1.19)	<0.001	7160 (5980 to 8040)	0.18 (0.15 to 0.2)	−0.87 (−1.18 to −0.56)	<0.001
Ovarian cancer	85,750 (75,170 to 95,090)	2.17 (1.9 to 2.41)	0.43 (0.24 to 0.62)	<0.001	25,260 (22,280 to 27,860)	0.64 (0.56 to 0.71)	0.17 (−0.03 to 0.36)	0.099
Prostate cancer	17,870 (15,620 to 19,480)	0.45 (0.4 to 0.49)	0.75 (0.46 to 1.05)	<0.001	2860 (2260 to 3240)	0.07 (0.06 to 0.08)	−0.19 (−0.31 to −0.07)	0.003
Testicular cancer	76,360 (73,290 to 79,920)	1.93 (1.86 to 2.02)	1.37 (0.96 to 1.78)	<0.001	7390 (6960 to 7840)	0.19 (0.18 to 0.2)	−0.04 (−0.24 to 0.16)	0.681
Kidney cancer	52,630 (49,670 to 55,820)	1.33 (1.26 to 1.41)	0.68 (0.55 to 0.8)	<0.001	10,980 (10,260 to 11,700)	0.28 (0.26 to 0.3)	−0.22 (−0.39 to −0.06)	0.007
Bladder cancer	31,050 (28,340 to 34,320)	0.79 (0.72 to 0.87)	−0.26 (−0.49 to −0.03)	0.027	6330 (5730 to 7000)	0.16 (0.15 to 0.18)	−1.31 (−1.42 to −1.2)	<0.001
Central nervous system cancer	97,460 (83,230 to 113,390)	2.47 (2.11 to 2.87)	0.35 (0.26 to 0.44)	<0.001	54,850 (46,390 to 64,940)	1.39 (1.17 to 1.64)	−0.34 (−0.46 to −0.22)	<0.001
Eye cancer	7190 (5100 to 9780)	0.18 (0.13 to 0.25)	0.86 (0.78 to 0.94)	<0.001	1290 (930 to 1720)	0.03 (0.02 to 0.04)	0.57 (0.46 to 0.68)	<0.001
Peripheral nervous system cancer	2000 (1660 to 2380)	0.05 (0.04 to 0.06)	1.09 (0.76 to 1.42)	<0.001	1050 (920 to 1150)	0.03 (0.02 to 0.03)	0.97 (0.82 to 1.11)	<0.001
Thyroid cancer	96,290 (84,000 to 110,260)	2.44 (2.13 to 2.79)	1.7 (1.6 to 1.79)	<0.001	5500 (4690 to 6410)	0.14 (0.12 to 0.16)	0.24 (0.15 to 0.34)	<0.001
Mesothelioma	3080 (2770 to 3400)	0.08 (0.07 to 0.09)	0.12 (0.00 to 0.24)	0.049	2530 (2260 to 2810)	0.06 (0.06 to 0.07)	0.1 (−0.02 to 0.22)	0.095
Hodgkin lymphoma	35,590 (28,930 to 42,840)	0.9 (0.73 to 1.08)	−1.1 (−1.25 to −0.96)	<0.001	11,490 (8150 to 15,310)	0.29 (0.21 to 0.39)	−1.64 (−1.73 to −1.55)	<0.001
Non-Hodgkin lymphoma	116,610 (107,010 to 126,120)	2.95 (2.71 to 3.19)	0.29 (0.05 to 0.54)	0.017	39,130 (35,270 to 43,700)	0.99 (0.89 to 1.11)	−0.49 (−0.57 to −0.41)	<0.001
Multiple myeloma	9770 (7980 to 11,260)	0.25 (0.2 to 0.29)	0.84 (0.7 to 0.99)	<0.001	6030 (4890 to 7140)	0.15 (0.12 to 0.18)	0.62 (0.51 to 0.72)	<0.001
Leukemia	94,730 (77,220 to 105,830)	2.4 (1.96 to 2.68)	−0.87 (−1.07 to −0.68)	<0.001	66,690 (54,520 to 74,800)	1.69 (1.38 to 1.89)	−1.4 (−1.6 to −1.2)	<0.001
Other cancers	74,620 (65,900 to 81,340)	1.89 (1.67 to 2.06)	0.53 (0.36 to 0.7)	<0.001	30,700 (26,950 to 33,740)	0.78 (0.68 to 0.85)	−0.55 (−0.77 to −0.33)	<0.001

Abbreviations: CI, confidence interval; MASH, metabolic dysfunction-associated steatohepatitis; UI, uncertainty interval.

## Data Availability

The data analyzed in this study were obtained from the Global Burden of Disease (GBD) study in 2021 and can be retrieved using the Global Health Data Exchange (GHDx) query tool http://ghdx.healthdata.org/gbd-results-tool (accessed on 22 October 2024), which is maintained by the Institute for Health Metrics and Evaluation (IHME).

## Supplementary Materials

The following supporting information can be downloaded at https://www.mdpi.com/article/10.3390/cancers17172766/s1. Material S1: Sociodemographic index of national and subnational based on the GBD 2021 study. Material S2: Overview of Global Burden of Disease Methodology. Table S1: List of International Classification of Diseases (ICD) codes mapped to the Global Burden of Disease cause list for cancer mortality data. Table S2: Incidence and death of early-onset cancer from 2019 to 2021, by region and SDI. Table S3: Incidence, death, age-standardized incidence rate, age-standardized death rate, and change from 2000 to 2021 of cancer in patients aged 15–49, by 5-year age group. Table S4: Incidence, age-standardized incidence rate, and change from 2000 to 2021 of cancer in patients aged 15–49 in females and males, by region and sociodemographic index. Table S5: Death, age-standardized death rate, and change from 2000 to 2021 of cancer in patients aged 15–49 in females and males, by region and sociodemographic index. Table S6: Incidence, age-standardized incidence rate, and change from 2000 to 2021 of cancer in patients aged 15–49, by country.

## Abbreviations

The following abbreviations are used in this manuscript:

APC	Annual percent change
ASDR	Age-standardized death rate
ASIR	Age-standardized incidence rate
ASRs	Age-standardized rates
CODEm	Cause of Death Ensemble model
CI	Confidence interval
CNS	Central Nervous System
GBD	Global Burden of Disease
GHDx	Global Health Data Exchange
ICD-10	The 10th revision of the International Statistical Classification of Diseases
IHME	Institute for Health Metrics and Evaluation
MIR	Mortality-to-incidence ratio
PNS	Peripheral Nervous System
SDI	Sociodemographic index
ST-GPR	Spatiotemporal Gaussian process regression
UIs	Uncertainty intervals
WHO	World Health Organization
