# Supplementary material for: Contemporary Changes in Global Trends in Early-Onset Cancer: Incidence and Mortality (2000–2021)
Source: Cancers (Basel). 2025 Aug 25;17(17):2766. doi: 10.3390/cancers17172766 (PMC12427225; doi:10.3390/cancers17172766)
Supplement: Supplementary file 1 [file cancers-17-02766-s001.zip › cancers-3704483-supplementary.pdf]

Supplementary Material 1: Sociodemographic index of national and subnational based on the GBD 2021 study.

Low SDI

Addis Ababa. **Afghanistan**. Afar. Amhara. Bauchi. **Benin**. Benishangul-Gumuz. Bihar, Rural. Borno. **Burkina Faso**. **Burundi**. Balochistan. **Côte d'Ivoire**. **Central African Republic**. **Chad**. **Democratic Republic of the Congo**. Dire Dawa. **Eritrea**. **Gambia**. Gambella. Gilgit-Baltistan. Gombe. **Guinea**. **Guinea-Bissau**. **Haiti**. Harari. Jharkhand, Rural. Jigawa. Kaduna. Kano. Katsina. Kebbi. Khyber Pakhtunkhwa. **Liberia**. **Madagascar**. **Malawi**. **Mali**. Madhya Pradesh, Rural. **Mozambique**. **Nepal**. **Niger**. Oromia. **Papua New Guinea**. **Rwanda**. **Senegal**. **Sierra Leone**. Sokoto. **Solomon Islands**. **Somalia**. Southern Nations, Nationalities, and Peoples. **South Sudan**. Taraba. **Timor-Leste**. Tigray. **Togo**. **Uganda**. **United Republic of Tanzania**. **Yemen**. Yobe. Zamfara.

Low Middle SDI

Acre. Adamawa. Akwa Ibom. Alagoas. Amazonas. Andhra Pradesh, Rural. **Angola**. Arunachal Pradesh, Rural. Assam, Rural. Azad Jammu & Kashmir. Bahia. **Bangladesh**. Baringo. Bayelsa. **Belize**. Bengkulu. Benue. **Bhutan**. Bihar, Urban. **Bolivia (Plurinational State of)**. Bomet. Bungoma. Busia. **Cabo Verde**. **Cambodia**. **Cameroon**. Ceará. Central Java. Central Sulawesi. Chhattisgarh, Rural. **Comoros**. **Congo**. Cross River. Delta. **Democratic People's Republic of Korea**. **Djibouti**. East Nusa Tenggara. Ebonyi. **Egypt**. Ekiti. **El Salvador**. Elgeyo-Marakwet. Embu. Enugu. **Eswatini**. Garissa. **Ghana**. Gorontalo. Guatemala. Gujarat, Rural. Gujarat, Rural. Haryana, Rural. Homa Bay. **Honduras**. Isiolo. Jammu & Kashmir and Ladakh, Rural. Kajiado. Kakamega. Karnataka, Rural. Kericho. Kiambu. Kilifi. **Kiribati**. Kirinyaga. Kisii. Kisumu. Kitui. Kogi. Kwale. Kwara. **Kyrgyzstan**. Laikipia. Lampung. Lamu. **Lao People's Democratic Republic**. **Lesotho**. Machakos. Maharashtra, Rural. Makueni. Maluku. Mandera. Manipur, Rural. Maranhão. Marsabit. **Marshall Islands**. **Mauritania**. Meghalaya, Rural. Meru. **Micronesia (Federated States of)**. Migori. Mizoram, Rural. Mombasa. **Mongolia**. **Morocco**. Murang'a. **Myanmar**. Nagaland, Rural. Nairobi. Nakuru. **Namibia**. Nandi. Narok. Nasarawa. **Nicaragua**. North Maluku. Nyamira. Nyandarua. Nyeri. Odisha, Rural. Ogun. Ondo. Other Union Territories, Rural. Oyo. Paraíba. Pará. Pernambuco. Piauí. Plateau. Punjab. Punjab, Rural. Rajasthan, Rural. Rio Grande do Norte. Rondônia. Roraima. Samburu. **Samoa**. **Sao Tome and Principe**. Sergipe. Siaya. Sikkim, Rural. Sindh. Southeast Sulawesi. **Sudan**. Taita Taveta. **Tajikistan**. Tamil Nadu, Rural. Tana River. Telangana, Rural. Tharaka Nithi. Tibet. Tocantins. Trans Nzoia. Tripura, Rural. Turkana. **Tuvalu**. Uasin Gishu. Uttarakhand, Rural. Uttar Pradesh, Rural. **Vanuatu**. **Venezuela (Bolivarian Republic of**). Vihiga. Wajir. West Bengal, Rural. West Kalimantan. West Nusa Tenggara. West Pokot. West Sulawesi. **Zambia**. **Zimbabwe**.

Middle SDI

Abia. Abra. Aceh. Aguascalientes. Agusan Del Norte. Agusan Del Sur. Aklan. **Albania**. Albay. Alborz. **Algeria**. Amapá. Anambra. Andhra Pradesh, Urban. Anhui. Antique. Apayao. Ardebil. **Armenia**. Arunachal Pradesh, Urban. Assam, Urban. **Aurora**. **Azerbaijan**. Baja California. Baja California Sur. Bali. Bangka-Belitung Islands. Banten. Basilan. Bataan. Batanes. Batangas. Benguet. Biliran. Bohol. **Botswana**. Bukidnon. Bulacan. Bushehr. Cagayan. Camarines Norte. Camarines Sur. Camiguin. Campeche. Capiz. Catanduanes. Cavite. Cebu. Central Kalimantan. Chahar Mahaal and Bakhtiari. Chhattisgarh, Urban. Chiapas. Chihuahua. Coahuila. Colima. **Colombia**. **Costa Rica**. Cotabato (North Cotabato). **Cuba**. Davao de Oro. Davao Del Norte. Davao Del Sur. Davao Occidental. Davao Oriental. Delhi, Rural. Dinagat Islands. **Dominican Republic**. Durango. East Azarbayejan. East Java. Eastern Cape. Eastern Samar. **Ecuador**. Edo. **Equatorial Guinea**. Espírito Santo. Fars. FCT (Abuja). **Fiji**. Free State. **Gabon**. Gansu. Gauteng. Gilan. Goa, Rural. Goiás. Golestan. **Grenada**. Guanajuato. Guangxi. Guerrero. Guimaras. Guizhou. Gujarat, Urban. **Guyana**. Hainan. Hamadan. Haryana, Urban. Hebei. Henan. Hidalgo. Himachal Pradesh, Rural. Hormozgan. Hubei. Hunan. Ifugao. Ilam. Ilocos Norte. Ilocos Sur. Iloilo. Imo. **Iraq**. Isabela. Isfahan. Islamabad Capital Territory. Jalisco. **Jamaica**. Jambi. Jammu & Kashmir and Ladakh, Urban. Jharkhand, Urban. Jiangxi. Kalinga. Karnataka, Urban. Kerala. Kerala, Rural. Kerala, Urban. Kerman. Kermanshah. Khorasan-e-Razavi. Khuzestan. Kohgiluyeh and Boyer-Ahmad. Kurdistan. KwaZulu-Natal. La Union. Lagos. Laguna. Lanao Del Norte. Lanao Del Sur. Leyte. Limpopo. Lorestan. Madhya Pradesh, Urban. Maguindanao. Maharashtra, Urban. **Maldives**. Manipur, Urban. Marinduque. Markazi. Masbate. Mato Grosso. Mato Grosso do Sul. Mazandaran. Meghalaya, Urban. Mexico City. Michoacán. Michoacán de Ocampo. Minas Gerais. Misamis Occidental. Misamis Oriental. Mizoram, Urban. Morelos. Mountain Province. Mpumalanga. **México**. Nagaland, Urban. National Capital Region. **Nauru**. Nayarit. Negros Occidental. Negros Oriental. Ningxia. North Khorasan. North Sulawesi. North Sumatra. North-West. Northern Cape. Northern Samar. Nueva Ecija. Nueva Vizcaya. Nuevo León. Oaxaca. Occidental Mindoro. Odisha, Urban. Oriental Mindoro. Osun. Other Union Territories, Urban. Palawan. **Palestine**. Pampanga. **Panama**. Pangasinan. Papua. **Paraguay**. Paraná. **Peru**. Puebla. Punjab, Urban. Qazvin. Qinghai. Qom. Querétaro. Quezon. Quintana Roo. Quirino. Rajasthan, Urban. Rio de Janeiro. Rio Grande do Sul. Rivers. Rizal. Romblon. **Saint Lucia. Saint Vincent and the Grenadines**. Samar (Western Samar). San Luis Potosí. Santa Catarina. Sarangani. Semnan. Shanxi. Sichuan. Sikkim. Sikkim, Urban. Sinaloa. Siquijor. Sistan and Baluchistan. Sonora. Sorsogon. South Cotabato. South Kalimantan. South Khorasan. South Sulawesi. South Sumatra. Southern Leyte. **Sri Lanka**. Sultan Kudarat. Sulu. Surigao Del Norte. Surigao Del Sur. **Suriname**. **Syrian Arab Republic**. São Paulo. Tabasco. Tamaulipas. Tamil Nadu. Tamil Nadu, Urban. Tarlac. Tawi-Tawi. Tehran. Telangana. Telangana, Urban. **Thailand**. Tlaxcala. Tokelau. **Tonga**. Tripura. Tripura, Urban. **Tunisia**. **Turkmenistan**. Uttar Pradesh. Uttar Pradesh, Urban. **Uzbekistan**. Veracruz de Ignacio de la Llave. **Viet Nam**. West Azarbayejan. West Bengal. West Bengal, Urban. West Java. West Papua. West Sumatra. Western Cape. Xinjiang. Yazd. Yogyakarta. Yucatán. Yunnan. Zacatecas. Zambales. Zamboanga Del Norte. Zamboanga Del Sur. Zamboanga Sibugay. Zanjan.

High Middle SDI

Abruzzo. Altai Krai. American Samoa. Amur Oblast. **Antigua and Barbuda**. **Argentina**. Arkhangelsk oblast. Arkhangelsk oblast without Nenets autonomous district. Astrakhan Oblast. **Bahamas**. **Bahrain**. **Barbados**. Basilicata. **Belarus**. Belgorod Oblast. **Bosnia and Herzegovina**. **Brunei Darussalam**. Bryansk Oblast. **Bulgaria**. Calabria. Campania. Chechen Republic. Chelyabinsk oblast. **Chile**. Chongqing. Chukotka Autonomous Area. Chukotka Autonomous Okrug. Chuvash Republic. **Cook Islands**. **Croatia**. Delhi, Urban. Distrito Federal. **Dominica**. East Kalimantan. Emilia-Romagna. Friuli-Venezia Giulia. Fujian. **Georgia**. Goa, urban. **Greece**. **Guam**. Guangdong. Heilongjiang. Himachal Pradesh, urban. **Hungary**. Inner Mongolia. Irkutsk Oblast. **Israel**. Ivanovo Oblast. Jakarta. Jewish Autonomous Oblast. Jiangsu. Jilin. **Jordan**. Kabardino-Balkar Republic. Kaliningrad Oblast. Kaluga Oblast. Kamchatka Krai. Karachay-Cherkess Republic. **Kazakhstan**. Kemerovo Oblast. Khabarovsk Krai. Khanty-Mansi autonomous area. Kirov Oblast. Komi Republic. Kostroma Oblast. Krasnodar Krai. Krasnoyarsk Krai. Kurgan Oblast. Kursk Oblast. Lazio. **Lebanon**. Leningrad Oblast. Liaoning. **Libya**. Liguria. Lipetsk Oblast. Lipetzk oblast. Lombardia. Magadan Oblast. **Malaysia**. **Malta**. Marche. **Mauritius**. Molise. **Montenegro**. Moscow City. Moscow Oblast. Murmansk Oblast. Nenets autonomous district. **Niue**. Nizhny Novgorod Oblast. North Kalimantan. **North Macedonia**. Northern Mariana Islands. Novgorod Oblast. Novosibirsk Oblast. **Oman**. Omsk Oblast. Orenburg Oblast. Oryol Oblast. **Palau**. Penza Oblast. Perm Krai. Piemonte. **Portugal**. Primorsky Krai. Provincia autonoma di Bolzano. Provincia autonoma di Trento. Pskov Oblast. Puglia. Republic of Adygeya. Republic of Altai. Republic of Bashkortostan. Republic of Buryatia. Republic of Crimea. Republic of Dagestan. Republic of Ingushetia. Republic of Kalmykia. Republic of Karelia. Republic of Khakassia. Republic of Mari El. **Republic of Moldova**. Republic of Mordovia. Republic of North Ossetia-Alania. Republic of Sakha (Yakutia). Republic of Tatarstan. Republic of Tuva. Riau. Riau Islands. **Romania**. Rostov Oblast. Ryazan Oblast. **Saint Kitts and Nevis**. Saint Petersburg. Sakhalin Oblast. Samara Oblast. Saratov Oblast. Sardegna. **Serbia**. Sevastopol. **Seychelles**. Shaanxi. Shandong. Sicilia. **Slovakia**. Smolensk Oblast. **Spain**. Stavropol Krai. Sverdlovsk Oblast. Tambov Oblast. Tomsk Oblast. Toscana. **Trinidad and Tobago** Tula Oblast. Tver Oblast. Tyumen Oblast. Tyumen oblast without autonomous areas. **Türkiye**. Udmurt Republic. **Ukraine**. **Ukraine (without Crimea & Sevastopol)**. Ulyanovsk Oblast. Umbria. **Uruguay**. Uttarakhand. Uttarakhand, Urban. Valle d'Aosta. Veneto. Vladimir Oblast. Volgograd Oblast. Vologda Oblast. Voronezh Oblast. Yamalo-Nenets autonomous area. Yamalo-Nenets Autonomous Okrug. Yaroslavl Oblast. Zabaikalsk kray. Zhejiang.

High SDI

Agder. Aichi. Akita. Alabama. Alaska. **Andorra**. Aomori. Arizona. Arkansas. **Australia**. **Austria**. Barking and Dagenham. Barnet. Barnsley. Bath and North East Somerset. Bedford. Beijing. **Belgium**. Bermuda. Bexley. Birmingham. Blackburn with Darwen. Blackpool. Bolton. Bournemouth. Bracknell Forest. Bradford. Brent. Brighton and Hove. Bristol, City of. Bromley. Buckinghamshire. Bury. Calderdale. California. Cambridgeshire. Camden. **Canada**. Central Bedfordshire. Cheshire East. Cheshire West and Chester. Chiba. Colorado. Connecticut. Cornwall. County Durham. Coventry. Croydon. Cumbria. **Cyprus**. **Czechia**. Darlington. Delaware. **Denmark**. Derby. Derbyshire. Devon. District of Columbia. Dolnośląskie. Doncaster. Dorset. Dudley. Ealing. East Riding of Yorkshire. East Sussex. Ehime. Enfield. Essex. **Estonia**. **Finland**. Florida. **France**. Fukui. Fukuoka. Fukushima. Gateshead. **Georgia. Germany**. Gifu. Gloucestershire. **Greenland**. Greenwich. Gunma. Hackney. Halton. Hammersmith and Fulham. Hampshire. Haringey. Harrow. Hartlepool. Havering. Hawaii. Herefordshire, County of. Hertfordshire. Hillingdon. Hiroshima. Hokkaidō. Hong Kong. Hong Kong Special Administrative Region of China. Hounslow. Hyōgo. Ibaraki. **Iceland**. Idaho. Illinois. Indiana. Innlandet. Iowa. **Ireland**. Ishikawa. Isle of Wight. Islington. Iwate. Kagawa. Kagoshima. Kanagawa. Kansas. Kensington and Chelsea. Kent. Kentucky. Kingston upon Hull, City of. Kingston upon Thames. Kirklees. Knowsley. Kujawsko-Pomorskie. Kumamoto. **Kuwait**. Kyoto. Kōchi. Lambeth. Lancashire. **Latvia**. Leeds. Leicester. Leicestershire. Lewisham. Lincolnshire. **Lithuania**. Liverpool. Łódzkie. Louisiana. Lubelskie. Lubuskie. Luton. **Luxembourg**. Macao. Macao Special Administrative Region of China. Maine. Manchester. Maryland. Massachusetts. Mazowieckie. Małopolskie. Medway. Merton. Michigan. Middlesbrough. Mie. Milton Keynes. Minnesota. Mississippi. Missouri. Miyagi. Miyazaki. **Monaco**. Montana. Møre og Romsda. Nagano. Nagasaki. Nara. Nebraska. **Netherlands**. Nevada. New Hampshire. New Jersey. New Mexico. New York. **New Zealand**. New Zealand Maori population. New Zealand non-Maori population. Newcastle upon Tyne. Newham. Niigata. Nordland. Norfolk. North Carolina. North Dakota. North East Lincolnshire. North Lincolnshire. North Somerset. North Tyneside. North Yorkshire. Northamptonshire. **Northern Ireland**. Northumberland. Nottingham. Nottinghamshire. Ohio. Oita. Okayama. Okinawa. Oklahoma. Oldham. Opolskie. Oregon. Osaka. Oslo. Oxfordshire. Pennsylvania. Peterborough. Plymouth. Podkarpackie. Podlaskie. Pomorskie. Poole. Portsmouth. Puerto Rico. **Qatar**. Reading. Redbridge. Redcar and Cleveland. **Republic of Korea**. Rhode Island. Richmond upon Thames. Rochdale. Rogaland. Rotherham. Rutland. Saga. Saitama. Salford. **San Marino**. Sandwell. **Saudi Arabia**. **Scotland**. Sefton. Shanghai. Sheffield. Shiga. Shimane. Shizuoka. Shropshire. **Singapore**. Śląskie. Slough. **Slovenia**. Solihul. Somerset. South Carolina. South Dakota. South Gloucestershire. South Tyneside. Southampton. Southend-on-Sea. Southwark. St Helens. Staffordshire. Stockholm. Stockport. Stockton-on-Tees. Stoke-on-Trent. Suffolk. Sunderland. Surrey. Sutton. **Sweden**. Sweden except Stockholm. Świętokrzyskie. Swindon. **Switzerland**. **Taiwan (Province of China)**. Tameside. Telford and Wrekin. Tennessee. Texas. Thurrock. Tianjin. Tochigi. Tokushima. Tokyo. Torbay. Tottori. Tower Hamlets. Toyama. Trafford. Troms og Finnmark. Trøndelag. **United Arab Emirates**. United States Virgin Islands. Utah. Vermont. Vestfold og Telemark. Vestland. Viken. Virginia. Wakayama. Wakefield. **Wales**. Walsall. Waltham Forest. Wandsworth. Warmińsko-Mazurskie. Warrington. Warwickshire. Washington. West Berkshire. West Sussex. West

Virginia. Westminster. Wielkopolskie. Wigan. Wiltshire. Windsor and Maidenhead. Wirral. Wisconsin. Wokingham. Wolverhampton. Worcestershire. Wyoming. Yamagata. Yamaguchi. Yamanashi. York. Zachodniopomorskie.

The subnational sociodemographic Index can be accessed at https://doi.org/10.1016/S0140-6736(24)00757-8

Supplementary Material 2. Overview of Global Burden of Disease Methodology

1. Overview

The GBD 2021 estimates mortality and causes of mortality for diseases in 204 countries and territories using a standard methodological approach.

The GBD 2021 uses a variety of data input sources, including surveys, censuses, vital statistics, and other health-related data sources to estimate mortality rates. The input sources are available via the interactive citation tool using the Global Health Data Exchange (GHDx; http://ghdx.healthdata.org/). The user can view and access GHDx records for input sources and export a comma-separated value (CSV) file comprising metadata, citations, and information on where data were used in GBD. Citations for specific GBD components, causes and risks, and locations can be located via this tool. Moreover, the GBD allows the visualisation of its results online. All GBD 2021 online data visualisations are available at https://vizhub.healthdata.org/gbd-compare/, which offers results for all GBD health metrics. The core summary GBD 2021 results comprising mortality data can be obtained in tabular form with the GBD's data download tool, available at http://ghdx.healthdata.org/gbd-results-tool. Users must provide an email address, depending on download size, where a download location will be provided to them when the files are ready.

2. Causes of Death Database

Data sources for causes of death were retrieved from vital registration systems, verbal autopsies, and other surveillance systems from 2000-2021. The available data on the cause of death (CoD) are standardised based on the International Classification of Diseases (ICD) 9 and 10 code mapping and pooled into a single database used to generate cause-specific mortality estimates by sex, year, regional geography, and age. The CoD database includes seven data sources, including vital registration (VR), verbal autopsy (VA), sibling history, and survey/census. There is no need to use any other data source in countries with complete VR systems. However, less than half of the global population has deaths captured in a VR system. Hence, for these countries with incomplete VR systems, vital statistics for causes of death may be supplemented with other data types[3]. Data inputs used to generate the estimates are found at http://ghdx.healthdata.org/gbd-2021/data-input-sources.

Most of the CoD data is VR data obtained from WHO Mortality Database, a summation of data submitted to WHO by individual countries. VR is obtained from country-specific mortality databases operated by official offices. Whenever possible, each cause is coded directly to the most detailed CoD, whilst cause codes in the data tabulated by ICD are coded to aggregated cause groups. Many countries have adopted the ICD Tabulation lists. The ICD tabulation lists include the ICD-9 Basic Tabulation List, the ICD-10 Mortality Tabulation, the Russia Tabulation, and the India Medical Certification of Cause of Death. Sample registration systems are increasingly used in several countries, such as Indonesia and India. In those without VR systems, VA studies are a viable data source to inform CoD. Data are retrieved by trained interviewers who utilise a standardised questionnaire to ask relatives about the signs, symptoms, and demographic characteristics of recently deceased family members. CoD is assigned based on the answers to the questionnaires. Hence, VA data are highly heterogeneous as studies adopt different instruments, cause lists (from single causes to complete ICD-cause lists), methods for assigning CoD, recall periods, and age groups. Cultural differences may also play a part in affecting the interpretation of specific questions. When mapping to the GBD cause, CoD validity might be considered. VAs are likely less accurate for causes requiring medical certification, such as diabetes, than assigning CoD to road injury or homicide.

3. Steps in Data Input

Step 1: Standardise input data

Diverse formats such as mortality databases, literature reviews, and reports were used to collect the input data for the cause of death (CoD) database. Usable data sources must demonstrate an apparent sample size of the number of deaths in the population and exhaustive cause lists. The data cleaning process remains complex and differs across data sources; for instance, minimal effort is needed to standardise the data into a consistent structure for VR microdata with location, age, sex, year, and ICD-coded cause of death. The data are subsequently assigned source identifiers to be linked to the GHDx and cited appropriately. Aggregate sex and age categories are then identified for sex-age splitting. Documentation from the source is reviewed to ensure that the population is representative of the location or only a subgroup of the population within that region. Diagnostics were also reviewed at the final stage to avoid sending cleaning errors downstream. All death totals were compared with the sum of cause-specific deaths to ensure that all observed deaths were accounted for and the sample size was complete.

The CoD in tabulated VR data were then compiled into aggregated groups, and some were mapped directly to GBD causes, while others were not informative and thus, cannot be mapped to them. The aggregated causes were mapped and split into ICD detail causes or targets based on the ICD groupings within the aggregated causes. The proportions of deaths from nearby countries with the super-region were used to fill in data gaps, as they tended to have similar CoD trends. Global proportions were used for any cause and demographic group for which ICD-detail was lacking.

Step 2: Map to GBD cause list

GBD 2021 used 439 maps to ensure that the cause list from the input data matched the GBD cause list. Examples of the largest and most widely accepted maps used were those of the ICD9 and ICD10 VR data. The mapping process allowed for comparing various data sources across demographic groups.

Step 3: Split Age-sex groups

Some input sources, particularly the VA studies, reported death for a wide range of age groups with different intervals. These different age intervals were then mapped to the GBD standard set of age groups. When the input source contains death due to impossible causes for the given sex and age, it was then redistributed proportionally to all causes.

Step 4: Redistribute

To improve the comparability of the data for CoD, redistribution of uninformative codes, or garbage codes, is needed. Garbage codes to which deaths were assigned should not be considered as the underlying CoD (for example, "heart failure", "ill-defined cancer site"). For each redistribution package, we defined the "universe" of data as all deaths coded to either the package's garbage codes or the package's redistribution targets for each country, year, age, and sex. A regression was run separately for each target group and sex.

Step 5: Correct post-redistribution problems

In this step, data were checked to ensure that the cause list at this point is reasonable given the original data source and how the CoD was assigned. There were two primary corrections that were applied – 1) any cause that is purely an artifact of the redistribution machinery targeting too precise a cause is aggregated up to the parent causes; 2) a "bridge map" is performed over a specific set of sources to ensure that they do not contain causes that could not be reliably determined by the methods used.

Step 6: Drop VR country years or mark them as non-representative

Lozano and colleagues describe the negative impact of low-completeness VR data on CoD modelling for GBD 2010. For GBD 2021, VR location-years with completeness less than 50% were dropped, and completeness between 50-69% was marked as non-representative. Moreover, any country-year with multiple mortalities registered to major garbage codes greater than 50% of the registered mortality was dropped.

Step 7: Aggregate causes

In this step, the cause list was organised into four levels in a top-down hierarchical format. For example, deaths are classified into three broad groups (Level 1 causes) that include "communicable, maternal, neonatal, nutritional diseases", "non-communicable diseases", and "injuries". Within the Level 1 group of non-communicable diseases is the Level 2 cause "diabetes and kidney diseases," which aggregates the Level 3 causes "Diabetes mellitus" and "chronic kidney disease". "Diabetes mellitus" further aggregates the level 4 causes "Diabetes mellitus type 1" and "Diabetes mellitus type 2"; whilst "chronic kidney disease" aggregates give level 4 causes of "chronic kidney disease due to diabetes type 1", "chronic kidney disease due to diabetes type 2", "Hypertensive chronic kidney disease", "glomerulonephritis chronic kidney disease" and "other chronic kidney disease". This example demonstrates that the mortality estimate for a parent cause in the hierarchy represents the sum of the deaths due to the causes under that rubric. The parent Level 3 cause estimate includes deaths mapped directly to the parent and any Level 4 sub-causes.

Step 8: Application of noise reduction algorithm

Bayesian noise reduction algorithm was used to deal with zero counts in VR and VA for a specific age group in a specific year. Here, we assume a normal prior and a normal data likelihood. Estimating normal prior for the given list of countries was done by running a Poisson regression to estimate the number of deaths due to a specific cause and sex with dummy variables for year and age.

Step 9: Identify outliers in the cause of death

As death rates for CoD tend to have a stable age pattern that does not change rapidly over time in a large population, it is fair to assume a relatively stable pattern in death rates for all causes. Rare exceptions to this include epidemic diseases and specific types of injuries. These outliers have been corrected using the noise reduction process as mentioned in step 8. Identifying outliers occurs before the models' finalisation for each cause. This is based on the judgment of the modeller and senior faculty, and these outlier decisions are reversible and may be revisited.

4. Causes of Death Modelling Methods

To estimate mortality causes, GBD uses the Cause of Death Ensemble model (CODEm), which combines results from different statistical models weighted based on the out-of-sample predictive validity. The CODEm relies on four key components: First, all available data were identified and gathered to be used in the modelling process. Although these available data may differ in quality, all these data contain some signal of the actual epidemiological process. Second, diverse plausible models are implemented to obtain well-documented associations in the estimates. Using a wide range of individual models to create a predictive ensemble model has outperformed techniques using only a single model both in CoD estimation and in more general prediction applications. Third, the out-of-sample predictive validity is assessed for all individual models, which are then ranked for use in the ensemble modelling stage. Fourth, differently weighted combinations of individual models are examined to select the ensemble model with the highest out-of-sample predictive validity.

A range of plausible statistical models was developed for each cause as several factors may co-vary with any of the CoD. In the CODEm framework, four groups of statistical models are utilized: 1) linear mixed effects regression (LMER) models of the natural log of the cause-specific death rate, 2) LMER models of the logit of the cause fraction, 3) spatiotemporal Gaussian process regression (ST-GPR) models of the natural logarithm of the cause-specific death rate, and 4) SR-GPR models of the logit of the cause fraction. The component models are weighted accordingly to their predictive validity rank to determine their contribution to the ensemble estimate. A set of ensemble models is then determined using the weights.

After the weighting scheme is decided, 1000 draws are created for the final ensemble, with the number of draws contributed by each model proportional to its weight. Mortality estimates were scaled with other causes of death to 100% of all-cause death estimates within each age, sex, year, and location. The estimate for each mortality cause is the mean of 1000 draws from the set of best-performing models. 95% uncertainty intervals (UIs) were calculated for all estimates to reflect the 25th and 95th percentile values of the 1000 draws. DALYs were estimated by the summation of years of life lost and years lost due to disability, which serves to quantify the extent of health loss related to specific diseases. Years of life lost were obtained by multiplying the estimated number of deaths by age with a standard life expectancy. In contrast, the multiplication of prevalence computed years lost due to disability by a disability weight, ranging from 0 to 1 where 0 is a state of full health, and 1 is death. Age-standardized rates per 100,000 population were also derived using the direct method from the GBD 2021 population estimate with five-year age groups. All estimates were reported with the corresponding 95% UIs.

The completed methodology from GBD 2021 can be found in the GBD 2021 capstone publication: 10.1016/S0140-6736(24)00367-2.

Supplementary Table 1: List of International Classification of Diseases (ICD) codes mapped to the Global Burden of Disease cause list for cancer mortality data

Copied from 10.1001/jamaoncol.2021.6987

Cause	ICD10	ICD9
Lip and oral cavity cancer	C00, C00.0, C00.1, C00.2, C00.3, C00.4, C00.5, C00.6, C00.8, C00.9, C01, C01.9, C02, C02.0, C02.1, C02.2, C02.3, C02.4, C02.8, C02.9, C03, C03.0, C03.1, C03.9, C04, C04.0, C04.1, C04.8, C04.9, C05, C05.0, C05.1, C05.2, C05.8, C05.9, C06, C06.0, C06.1, C06.2, C06.8, C06.80, C06.89, C06.9, C07, C07.0, C07.9, C08, C08.0, C08.1, C08.8, C08.9, D00.00, D00.01, D00.02, D00.03, D00.04, D00.05, D00.06, D00.07, D10.0, D10.1, D10.2, D10.3, D10.30, D10.39, D10.4, D10.5, D11, D11.0, D11.7, D11.9, D37.01, D37.02, D37.03, D37.030, D37.031, D37.032, D37.039, D37.04, D37.09	140, 140.0, 140.1, 140.2, 140.3, 140.4, 140.5, 140.6, 140.7, 140.8, 140.9, 141, 141.0, 141.1, 141.2, 141.3, 141.4, 141.5, 141.6, 141.8, 141.9, 142, 142.0, 142.1, 142.2, 142.3, 142.8, 142.9, 143, 143.0, 143.1, 143.8, 143.9, 144, 144.0, 144.1, 144.4, 144.8, 144.9, 145, 145.0, 145.1, 145.2, 145.3, 145.4, 145.5, 145.6, 145.8, 145.9, 210, 210.0, 210.1, 210.2, 210.3, 210.4, 210.5, 210.6, 235, 235.0
Nasopharynx cancer	C11, C11.0, C11.1, C11.2, C11.3, C11.8, C11.9, D00.08, D10.6, D37.05	147, 147.0, 147.1, 147.2, 147.3, 147.8, 147.9, 210.7, 210.8, 210.9
Other pharynx cancer	C09, C09.0, C09.1, C09.8, C09.9, C1, C10, C10.0, C10.1, C10.2, C10.3, C10.4, C10.8, C10.9, C12, C12.0, C12.9, C13, C13.0, C13.1, C13.2, C13.8, C13.9, D10.7	146, 146.0, 146.1, 146.2, 146.3, 146.4, 146.5, 146.6, 146.7, 146.8, 146.9, 148, 148.0, 148.1, 148.2, 148.3, 148.4, 148.5, 148.8, 148.9
Esophageal cancer	C15, C15.0, C15.1, C15.2, C15.3, C15.4, C15.5, C15.8, C15.9, D00.1, D13.0	150, 150.0, 150.1, 150.2, 150.3, 150.4, 150.5, 150.6, 150.7, 150.8, 150.9, 211, 211.0, 230.1
Stomach cancer	C16, C16.0, C16.1, C16.2, C16.3, C16.4, C16.5, C16.6, C16.7, C16.8, C16.9, D00.2, D13.1, D37.1	151, 151.0, 151.1, 151.2, 151.3, 151.4, 151.5, 151.6, 151.8, 151.9, 209.23, 209.63, 211.1, 230.2
Colon and rectum cancer	C18, C18.0, C18.1, C18.2, C18.3, C18.4, C18.5, C18.6, C18.7, C18.8, C18.9, C19, C19.0, C19.9, C2, C20, C20.0, C20.8, C20.9, C21, C21.0, C21.1, C21.2, C21.8, C21.9, D01.0, D01.1, D01.2, D01.3, D12, D12.0, D12.1, D12.2, D12.3, D12.4, D12.5, D12.6, D12.7, D12.8, D12.9, D37.3, D37.4, D37.5	153, 153.0, 153.1, 153.2, 153.3, 153.4, 153.5, 153.6, 153.7, 153.8, 153.9, 154, 154.0, 154.1, 154.2, 154.3, 154.4, 154.8, 154.9, 209.1, 209.10, 209.11, 209.12, 209.13, 209.14, 209.15, 209.16, 209.17, 209.5, 209.50, 209.51, 209.52, 209.53, 209.54, 209.55, 209.56, 209.57, 211.3, 211.4, 230.3, 230.4, 230.5, 230.6, 569.0, 569.43, 569.44, 569.84, 569.85
Liver cancer	C22, C22.0, C22.1, C22.3, C22.4, C22.5, C22.7, C22.8, D13.4	155, 155.0, 155.1, 155.3, 155.5, 155.9, 211.5
Gallbladder and biliary tract cancer	C23, C23.0, C23.9, C24, C24.0, C24.1, C24.4, C24.8, C24.9, D13.5	156, 156.0, 156.1, 156.2, 156.3, 156.8, 156.9, 209.65, 209.66, 209.67
Pancreatic cancer	C25, C25.0, C25.1, C25.2, C25.3, C25.4, C25.7, C25.8, C25.9, D13.6, D13.7	157, 157.0, 157.1, 157.2, 157.3, 157.4, 157.5, 157.7, 157.8, 157.9, 211.6, 211.7
Larynx cancer	C32, C32.0, C32.1, C32.2, C32.3, C32.8, C32.9, D02.0, D14.1, D38.0	161, 161.0, 161.1, 161.2, 161.3, 161.8, 161.9, 212.1, 231, 231.0, 235.6

Tracheal, bronchus, and lung cancer	C33, C33.0, C33.2, C33.9, C34, C34.0, C34.00, C34.01, C34.02, C34.1, C34.10, C34.11, C34.12, C34.2, C34.3, C34.30, C34.31, C34.32, C34.4, C34.7, C34.8, C34.80, C34.81, C34.82, C34.9, C34.90, C34.91, C34.92, D02.1, D02.2, D02.20, D02.21, D02.22, D02.3, D14.2, D14.3, D14.30, D14.31, D14.32, D38.1	162, 162.0, 162.1, 162.2, 162.3, 162.4, 162.5, 162.8, 162.9, 209.21, 209.61, 212.2, 212.3, 231.1, 231.2, 235.7
Malignant skin melanoma	C43, C43.0, C43.1, C43.10, C43.11, C43.12, C43.2, C43.20, C43.21, C43.22, C43.3, C43.30, C43.31, C43.39, C43.4, C43.5, C43.51, C43.52, C43.59, C43.6, C43.60, C43.61, C43.62, C43.7, C43.70, C43.71, C43.72, C43.8, C43.9, D03, D03.0, D03.1, D03.10, D03.11, D03.12, D03.2, D03.20, D03.21, D03.22, D03.3, D03.30, D03.39, D03.4, D03.5, D03.51, D03.52, D03.59, D03.6, D03.60, D03.61, D03.62, D03.7, D03.70, D03.71, D03.72, D03.8, D03.9, D22, D22.0, D22.1, D22.10, D22.11, D22.12, D22.2, D22.20, D22.21, D22.22, D22.3, D22.30, D22.39, D22.4, D22.5, D22.6, D22.60, D22.61, D22.62, D22.7, D22.70, D22.71, D22.72, D22.9, D23, D23.0, D23.1, D23.10, D23.11, D23.12, D23.2, D23.20, D23.21, D23.22, D23.3, D23.30, D23.39, D23.4, D23.5, D23.6, D23.60, D23.61, D23.62, D23.7, D23.70, D23.71, D23.72, D23.9	172, 172.0, 172.1, 172.2, 172.3, 172.4, 172.5, 172.6, 172.7, 172.8, 172.9
Non-melanoma skin cancer	D48.5	222.4
Non-melanoma skin cancer (squamous-cell carcinoma)	C44, C44.0, C44.00, C44.01, C44.02, C44.09, C44.1, C44.10, C44.101, C44.102, C44.109, C44.11, C44.111, C44.112, C44.119, C44.12, C44.121, C44.122, C44.129, C44.19, C44.191, C44.192, C44.199, C44.2, C44.20, C44.201, C44.202, C44.209, C44.21, C44.211, C44.212, C44.219, C44.22, C44.221, C44.222, C44.229, C44.29, C44.291, C44.292, C44.299, C44.3, C44.30, C44.300, C44.301, C44.309, C44.31, C44.310, C44.311, C44.319, C44.32, C44.320, C44.321, C44.329, C44.39, C44.390, C44.391, C44.399, C44.4, C44.40, C44.41, C44.42, C44.49, C44.5, C44.50, C44.500, C44.501, C44.509, C44.51, C44.510, C44.511, C44.519, C44.52, C44.520, C44.521, C44.529, C44.59, C44.590, C44.591, C44.599, C44.6, C44.60, C44.601, C44.602, C44.609, C44.61, C44.611, C44.612, C44.619, C44.62, C44.621, C44.622, C44.629, C44.69, C44.691, C44.692, C44.699, C44.7, C44.70, C44.701, C44.702, C44.709, C44.71, C44.711, C44.712, C44.719, C44.72, C44.721, C44.722, C44.729, C44.79, C44.791, C44.792, C44.799, C44.8, C44.80, C44.81, C44.82, C44.89, C44.9, C44.90, C44.91, C44.92, C44.99, D04, D04.0, D04.1, D04.10, D04.11, D04.12, D04.2, D04.20, D04.21,	173, 173.0, 173.00, 173.01, 173.02, 173.09, 173.1, 173.10, 173.11, 173.12, 173.19, 173.2, 173.20, 173.21, 173.22, 173.29, 173.3, 173.30, 173.31, 173.32, 173.39, 173.4, 173.40, 173.41, 173.42, 173.49, 173.5, 173.50, 173.51, 173.52, 173.59, 173.6, 173.60, 173.61, 173.62, 173.69, 173.7, 173.70, 173.71, 173.72, 173.79, 173.8, 173.80, 173.81, 173.82, 173.89, 173.9, 173.90, 173.91, 173.92, 173.99, 232, 232.0, 232.1, 232.2, 232.3, 232.4, 232.5, 232.6, 232.7, 232.8, 232.9, 238.2

	D04.22, D04.3, D04.30, D04.39, D04.4, D04.5, D04.6, D04.60, D04.61, D04.62, D04.7, D04.70, D04.71, D04.72, D04.8, D04.9, D49.2	
Breast cancer	C50, C50.0, C50.01, C50.011, C50.012, C50.019, C50.02, C50.021, C50.022, C50.029, C50.1, C50.11, C50.111, C50.112, C50.119, C50.12, C50.121, C50.122, C50.129, C50.2, C50.21, C50.211, C50.212, C50.219, C50.22, C50.221, C50.222, C50.229, C50.3, C50.31, C50.311, C50.312, C50.319, C50.32, C50.321, C50.322, C50.329, C50.4, C50.41, C50.411, C50.412, C50.419, C50.42, C50.421, C50.422, C50.429, C50.5, C50.51, C50.511, C50.512, C50.519, C50.52, C50.521, C50.522, C50.529, C50.6, C50.61, C50.611, C50.612, C50.619, C50.62, C50.621, C50.622, C50.629, C50.7, C50.8, C50.81, C50.811, C50.812, C50.819, C50.82, C50.821, C50.822, C50.829, C50.9, C50.91, C50.911, C50.912, C50.919, C50.92, C50.921, C50.922, C50.929, D05, D05.0, D05.00, D05.01, D05.02, D05.1, D05.10, D05.11, D05.12, D05.7, D05.8, D05.80, D05.81, D05.82, D05.9, D05.90, D05.91, D05.92, D24, D24.0, D24.1, D24.2, D24.9, D48.6, D48.60, D48.61, D48.62, D49.3	174, 174.0, 174.1, 174.2, 174.3, 174.4, 174.5, 174.6, 174.8, 174.9, 175, 175.0, 175.3, 175.9, 217, 217.0, 217.8, 233, 233.0, 238.3, 239.3, 610, 610.0, 610.1, 610.2, 610.3, 610.4, 610.8, 610.9
Cervical cancer	C53, C53.0, C53.1, C53.3, C53.4, C53.8, C53.9, D06, D06.0, D06.1, D06.7, D06.9, D26.0	180, 180.0, 180.1, 180.2, 180.3, 180.4, 180.5, 180.6, 180.8, 180.9, 219, 219.0, 233.1, 622.1, 622.10, 622.11, 622.12, 622.2, 622.7
Uterine cancer	C54, C54.0, C54.1, C54.2, C54.3, C54.4, C54.8, C54.9, D07.0, D07.1, D07.2, D26.1, D26.7, D26.9	182, 182.0, 182.1, 182.8, 182.9, 233.2
Ovarian cancer	C56, C56.0, C56.1, C56.2, C56.4, C56.9, D27, D27.0, D27.1, D27.9, D39.1, D39.10, D39.11, D39.12	183, 183.0, 220, 220.0, 220.9, 236.2
Prostate cancer	C61, C61.0, C61.9, D07.5, D29.1, D40.0	185, 185.0, 185.9, 222.2, 236.5
Testicular cancer	C62, C62.0, C62.00, C62.01, C62.02, C62.1, C62.10, C62.11, C62.12, C62.9, C62.90, C62.91, C62.92, D29.2, D29.20, D29.21, D29.22, D29.3, D29.30, D29.31, D29.32, D29.4, D29.7, D29.8, D40.1, D40.10, D40.11, D40.12, D40.7, D40.8	186, 186.0, 186.9, 222, 222.0, 222.3, 236.4
Kidney cancer	C64, C64.0, C64.1, C64.2, C64.4, C64.5, C64.6, C64.8, C64.9, C65, C65.0, C65.1, C65.2, C65.9, D30.0, D30.00, D30.01, D30.02, D30.1, D30.10, D30.11, D30.12, D41.0, D41.00, D41.01, D41.02, D41.1, D41.10, D41.11, D41.12	189.0, 189.1, 189.5, 189.6, 209.24, 209.64, 223, 223.0, 223.1, 236.91
Bladder cancer	C67, C67.0, C67.1, C67.2, C67.3, C67.4, C67.5, C67.6, C67.7, C67.8, C67.9, D09.0, D30.3, D41.4, D41.7, D41.8, D49.4	188, 188.0, 188.1, 188.2, 188.3, 188.4, 188.5, 188.6, 188.7, 188.8, 188.9, 223.3, 233.7, 236.7, 239.4
Brain and central nervous system cancer	C70, C70.0, C70.1, C70.5, C70.6, C70.9, C71, C71.0, C71.1, C71.2, C71.3, C71.4, C71.5, C71.6, C71.7, C71.8, C71.9, C72, C72.0, C72.1, C72.2, C72.20, C72.21, C72.22, C72.3, C72.30, C72.31, C72.32, C72.4, C72.40, C72.41, C72.42, C72.5, C72.50, C72.59, C72.8, C72.9	191, 191.0, 191.1, 191.2, 191.3, 191.4, 191.5, 191.6, 191.7, 191.8, 191.9, 192, 192.0, 192.1, 192.2, 192.3, 192.4, 192.8, 192.9

Thyroid cancer	C73, C73.0, C73.1, C73.2, C73.3, C73.4, C73.5, C73.8, C73.9, D09.3, D09.8, D34, D34.0, D34.9, D44.0	193, 193.0, 193.2, 193.9, 226, 226.0, 226.9
Mesothelioma	C45, C45.0, C45.1, C45.2, C45.3, C45.4, C45.5, C45.6, C45.7, C45.8, C45.9	NA
Hodgkin lymphoma	C81, C81.0, C81.00, C81.01, C81.02, C81.03, C81.04, C81.05, C81.06, C81.07, C81.08, C81.09, C81.1, C81.10, C81.11, C81.12, C81.13, C81.14, C81.15, C81.16, C81.17, C81.18, C81.19, C81.2, C81.20, C81.21, C81.22, C81.23, C81.24, C81.25, C81.26, C81.27, C81.28, C81.29, C81.3, C81.30, C81.31, C81.32, C81.33, C81.34, C81.35, C81.36, C81.37, C81.38, C81.39, C81.4, C81.40, C81.41, C81.42, C81.43, C81.44, C81.45, C81.46, C81.47, C81.48, C81.49, C81.5, C81.6, C81.7, C81.70, C81.71, C81.72, C81.73, C81.74, C81.75, C81.76, C81.77, C81.78, C81.79, C81.8, C81.9, C81.90, C81.91, C81.92, C81.93, C81.94, C81.95, C81.96, C81.97, C81.98, C81.99	201, 201.0, 201.00, 201.01, 201.02, 201.03, 201.04, 201.05, 201.06, 201.07, 201.08, 201.1, 201.10, 201.11, 201.12, 201.13, 201.14, 201.15, 201.16, 201.17, 201.18, 201.2, 201.20, 201.21, 201.22, 201.23, 201.24, 201.25, 201.26, 201.27, 201.28, 201.4, 201.40, 201.41, 201.42, 201.43, 201.44, 201.45, 201.46, 201.47, 201.48, 201.5, 201.50, 201.51, 201.52, 201.53, 201.54, 201.55, 201.56, 201.57, 201.58, 201.6, 201.60, 201.61, 201.62, 201.63, 201.64, 201.65, 201.66, 201.67, 201.68, 201.7, 201.70, 201.71, 201.72, 201.73, 201.74, 201.75, 201.76, 201.77, 201.78, 201.9, 201.90, 201.91, 201.92, 201.93, 201.94, 201.95, 201.96, 201.97, 201.98
Non-Hodgkin lymphoma	C83.7, C83.70, C83.71, C83.72, C83.73, C83.74, C83.75, C83.76, C83.77, C83.78, C83.79, C83.8, C82, C82.0, C82.00, C82.01, C82.02, C82.03, C82.04, C82.05, C82.06, C82.07, C82.08, C82.09, C82.1, C82.10, C82.11, C82.12, C82.13, C82.14, C82.15, C82.16, C82.17, C82.18, C82.19, C82.2, C82.20, C82.21, C82.22, C82.23, C82.24, C82.25, C82.26, C82.27, C82.28, C82.29, C82.3, C82.30, C82.31, C82.32, C82.33, C82.34, C82.35, C82.36, C82.37, C82.38, C82.39, C82.4, C82.40, C82.41, C82.42, C82.43, C82.44, C82.45, C82.46, C82.47, C82.48, C82.49, C82.5, C82.50, C82.51, C82.52, C82.53, C82.54, C82.55, C82.56, C82.57, C82.58, C82.59, C82.6, C82.60, C82.61, C82.62, C82.63, C82.64, C82.65, C82.66, C82.67, C82.68, C82.69, C82.7, C82.8, C82.80, C82.81, C82.82, C82.83, C82.84, C82.85, C82.86, C82.87, C82.88, C82.89, C82.9, C82.90, C82.91, C82.92, C82.93, C82.94, C82.95, C82.96, C82.97, C82.98, C82.99, C83,	200.2, 200.20, 200.21, 200.22, 200.23, 200.24, 200.25, 200.26, 200.27, 200.28, 200, 200.0, 200.00, 200.01, 200.02, 200.03, 200.04, 200.05, 200.06, 200.07, 200.08, 200.1, 200.10, 200.11, 200.12, 200.13, 200.14, 200.15, 200.16, 200.17, 200.18, 200.3, 200.30, 200.31, 200.32, 200.33, 200.34, 200.35, 200.36, 200.37, 200.38, 200.4, 200.40, 200.41, 200.42, 200.43, 200.44, 200.45, 200.46, 200.47, 200.48, 200.5, 200.50, 200.51, 200.52, 200.53, 200.54, 200.55, 200.56, 200.57, 200.58, 200.6, 200.60, 200.61, 200.62, 200.63, 200.64, 200.65, 200.66, 200.67, 200.68, 200.7, 200.70, 200.71, 200.72, 200.73,

	C83.0, C83.00, C83.01, C83.02, C83.03, C83.04, C83.05, C83.06, C83.07, C83.08, C83.09, C83.1, C83.10, C83.11, C83.12, C83.13, C83.14, C83.15, C83.16, C83.17, C83.18, C83.19, C83.2, C83.3, C83.30, C83.31, C83.32, C83.33, C83.34, C83.35, C83.36, C83.37, C83.38, C83.39, C83.4, C83.5, C83.50, C83.51, C83.52, C83.53, C83.54, C83.55, C83.56, C83.57, C83.58, C83.59, C83.6, C83.80, C83.81, C83.82, C83.83, C83.84, C83.85, C83.86, C83.87, C83.88, C83.89, C83.9, C83.90, C83.91, C83.92, C83.93, C83.94, C83.95, C83.96, C83.97, C83.98, C83.99, C84, C84.0, C84.00, C84.01, C84.02, C84.03, C84.04, C84.05, C84.06, C84.07, C84.08, C84.09, C84.1, C84.10, C84.11, C84.12, C84.13, C84.14, C84.15, C84.16, C84.17, C84.18, C84.19, C84.2, C84.3, C84.4, C84.40, C84.41, C84.42, C84.43, C84.44, C84.45, C84.46, C84.47, C84.48, C84.49, C84.5, C84.6, C84.60, C84.61, C84.62, C84.63, C84.64, C84.65, C84.66, C84.67, C84.68, C84.69, C84.7, C84.70, C84.71, C84.72, C84.73, C84.74, C84.75, C84.76, C84.77, C84.78, C84.79, C84.8, C84.9, C84.90, C84.91, C84.92, C84.93, C84.94, C84.95, C84.96, C84.97, C84.98, C84.99, C85, C85.0, C85.1, C85.10, C85.11, C85.12, C85.13, C85.14, C85.15, C85.16, C85.17, C85.18, C85.19, C85.2, C85.20, C85.21, C85.22, C85.23, C85.24, C85.25, C85.26, C85.27, C85.28, C85.29, C85.3, C85.4, C85.5, C85.6, C85.7, C85.8, C85.80, C85.81, C85.82, C85.83, C85.84, C85.85, C85.86, C85.87, C85.88, C85.89, C85.9, C85.90, C85.91, C85.92, C85.93, C85.94, C85.95, C85.96, C85.97, C85.98, C85.99, C86, C86.0, C86.1, C86.2, C86.3, C86.4, C86.5, C86.6, C96, C96.0, C96.1, C96.2, C96.3, C96.4, C96.5, C96.6, C96.7, C96.8, C96.9	200.74, 200.75, 200.76, 200.77, 200.78, 200.8, 200.80, 200.81, 200.82, 200.83, 200.84, 200.85, 200.86, 200.87, 200.88, 200.9, 202, 202.0, 202.00, 202.01, 202.02, 202.03, 202.04, 202.05, 202.06, 202.07, 202.08, 202.1, 202.10, 202.11, 202.12, 202.13, 202.14, 202.15, 202.16, 202.17, 202.18, 202.2, 202.20, 202.21, 202.22, 202.23, 202.24, 202.25, 202.26, 202.27, 202.28, 202.3, 202.30, 202.31, 202.32, 202.33, 202.34, 202.35, 202.36, 202.37, 202.38, 202.4, 202.40, 202.41, 202.42, 202.43, 202.44, 202.45, 202.46, 202.47, 202.48, 202.5, 202.50, 202.51, 202.52, 202.53, 202.54, 202.55, 202.56, 202.57, 202.58, 202.6, 202.60, 202.61, 202.62, 202.63, 202.64, 202.65, 202.66, 202.67, 202.68, 202.7, 202.70, 202.71, 202.72, 202.73, 202.74, 202.75, 202.76, 202.77, 202.78, 202.8, 202.80, 202.81, 202.82, 202.83, 202.84, 202.85, 202.86, 202.87, 202.88, 202.9, 202.90, 202.91, 202.92, 202.93, 202.94, 202.95, 202.96, 202.97, 202.98
Multiple myeloma	C88, C88.0, C88.00, C88.01, C88.1, C88.2, C88.20, C88.3, C88.4, C88.40, C88.7, C88.70, C88.71, C88.8, C88.9, C89, C90, C90.0, C90.00, C90.01, C90.02, C90.1, C90.10, C90.11, C90.12, C90.2, C90.20, C90.21, C90.22, C90.3, C90.30, C90.31, C90.32, C90.4, C90.5, C90.6, C90.7, C90.8, C90.9	203, 203.0, 203.00, 203.01, 203.02, 203.1, 203.10, 203.11, 203.12, 203.8, 203.80, 203.81, 203.82, 203.9
Leukemia	C91, C92	204, 205, 206
Acute lymphoid leukemia	C91.0, C91.00, C91.01, C91.02, C91.2, C91.3, C91.30, C91.31, C91.32, C91.6, C91.60, C91.61, C91.62	204.0, 204.00, 204.01, 204.02, 204.2, 204.20, 204.21, 204.22
Acute myeloid leukemia	C92.0, C92.00, C92.01, C92.02, C92.3, C92.30, C92.31, C92.32, C92.4, C92.40, C92.41, C92.42, C92.5, C92.50, C92.51, C92.52, C92.6, C92.60, C92.61, C92.62, C93.0, C93.00, C93.01, C93.02, C94.0, C94.00, C94.01, C94.02, C94.2, C94.20, C94.21, C94.22, C94.4, C94.40, C94.41, C94.42, C94.5	205.0, 205.00, 205.01, 205.02, 205.2, 205.20, 205.21, 205.22, 205.3, 205.30, 205.31, 205.32, 206.0, 206.00, 206.01, 206.02, 207.0, 207.00, 207.01, 207.02, 207.20, 207.8, 207.80, 207.81, 207.82

Chronic lymphoid leukemia	Custom mapping (see footnote)	Custom mapping (see footnote)
Chronic myeloid leukemia	C92.1, C92.10, C92.11, C92.12, C92.2, C92.20, C92.21, C92.22	205.1, 205.10, 205.11, 205.12
Other leukemia	C91.20, C91.70, C92.70, C92.80, C93, C93.1, C93.10, C93.11, C93.12, C93.3, C93.30, C93.31, C93.32, C93.8, C94, C94.1, C94.3, C94.30, C94.31, C94.32, C94.50, C94.6, C94.60, C94.7, C94.70, C94.8, C94.80, C94.81, C94.82, C95, C95.0, C95.00, C95.01, C95.02, C95.1, C95.10, C95.11, C95.12, C95.2, C95.4, C95.6, C95.7, C95.70, C95.9, C95.90, C95.91, C95.92	205.92, 206.1, 206.10, 206.11, 206.12, 207, 207.1, 207.10, 207.11, 207.12, 207.2, 207.21, 207.22, 207.9, 208, 208.0, 208.00, 208.01, 208.02, 208.1, 208.10, 208.11, 208.12, 208.2, 208.20, 208.21, 208.22, 208.4, 208.7, 208.8, 208.80, 208.81, 208.82, 208.9, 208.90, 208.91, 208.92
Other malignant neoplasms	C40, C40.0, C40.00, C40.01, C40.02, C40.1, C40.10, C40.11, C40.12, C40.2, C40.20, C40.21, C40.22, C40.3, C40.30, C40.31, C40.32, C40.8, C40.80, C40.81, C40.82, C40.9, C40.90, C40.91, C40.92, C41, C41.0, C41.01, C41.02, C41.1, C41.2, C41.3, C41.4, C41.5, C41.6, C41.7, C41.8, C41.9, C42.0, C42.1, C42.2, C42.3, C42.4, C69.0, C69.00, C69.01, C69.02, C69.1, C69.10, C69.11, C69.12, C69.3, C69.30, C69.31, C69.32, C69.4, C69.40, C69.41, C69.42, C69.5, C69.50, C69.51, C69.52, C69.6, C69.60, C69.61, C69.62, C69.7, C69.8, C69.80, C69.81, C69.82, C69.2, C69.20, C69.21, C69.22, C47, C47.0, C47.1, C47.10, C47.11, C47.12, C47.2, C47.20, C47.21, C47.22, C47.3, C47.4, C47.5, C47.6, C47.8, C47.9, C74.90, C17, C17.0, C17.1, C17.2, C17.3, C17.8, C17.9, C3, C30, C30.0, C30.1, C30.2, C30.3, C30.5, C30.8, C30.9, C31, C31.0, C31.1, C31.2, C31.3, C31.8, C31.9, C37, C37.0, C37.1, C37.2, C37.3, C37.9, C38, C38.0, C38.1, C38.2, C38.3, C38.4, C38.8, C4, C48, C48.0, C48.1, C48.2, C48.8, C48.9, C4A, C5, C51, C51.0, C51.1, C51.2, C51.8, C51.9, C52, C52.0, C52.9, C57, C57.0, C57.00, C57.01, C57.02, C57.1, C57.10, C57.11, C57.12, C57.2, C57.20, C57.21, C57.22, C57.3, C57.4, C57.7, C57.8, C58, C58.0, C58.9, C60, C60.0, C60.1, C60.2, C60.8, C60.9, C63, C63.0, C63.00, C63.01, C63.02, C63.1, C63.10, C63.11, C63.12, C63.2, C63.7, C63.8, C66, C66.0, C66.1, C66.2, C66.9, C68.0, C68.1, C68.8, C7, C75, C75.0, C75.1, C75.2, C75.3, C75.4, C75.5, C75.6, C75.8, D07.4, D09.2, D09.20, D09.21, D09.22, D13.2, D13.3, D13.30, D13.39, D14.0, D15, D15.0, D15.1, D15.2, D15.7, D15.9, D16, D16.0, D16.00, D16.01, D16.02, D16.1, D16.10, D16.11, D16.12, D16.2, D16.20,	170, 170.0, 170.1, 170.2, 170.3, 170.4, 170.5, 170.6, 170.7, 170.8, 170.9, 190, 190.0, 190.1, 190.2, 190.3, 190.4, 190.6, 190.7, 190.8, 190.5, 152, 152.0, 152.1, 152.2, 152.3, 152.4, 152.6, 152.8, 152.9, 158, 158.0, 158.3, 158.4, 158.5, 158.6, 158.8, 158.9, 160, 160.0, 160.1, 160.2, 160.3, 160.4, 160.5, 160.6, 160.8, 160.9, 163, 163.0, 163.1, 163.3, 163.5, 163.8, 163.9, 164, 164.0, 164.1, 164.2, 164.3, 164.8, 164.9, 181, 181.0, 181.9, 183.2, 183.3, 183.4, 183.5, 183.8, 184.0, 184.1, 184.2, 184.3, 184.4, 184.8, 187.1, 187.2, 187.3, 187.4, 187.5, 187.6, 187.7, 187.8, 189.2, 189.3, 189.4, 189.8, 194.1, 194.3, 194.4, 194.5, 194.6, 194.8, 209.0, 209.00, 209.01, 209.02, 209.03, 209.22, 209.25, 209.26, 209.27, 209.31, 209.32, 209.33, 209.34, 209.35, 209.36, 209.4, 209.40, 209.41, 209.42, 209.43, 211.2, 211.8, 212.0, 212.4, 212.5, 212.6, 212.7, 212.8, 213, 213.0, 213.1, 213.2, 213.3, 213.4, 213.5, 213.6, 213.7, 213.8, 213.9, 221.0, 221.1, 221.2, 221.8, 222.1, 222.8, 223.2, 223.8, 223.81, 223.89, 224, 224.0, 224.1, 224.2, 224.3, 224.4, 224.5, 224.6, 224.7, 224.8, 224.9, 225, 225.0, 225.1, 225.2, 225.3, 225.4, 225.8, 225.9, 227, 227.0,

	D16.21, D16.22, D16.3, D16.30, D16.31, D16.32, D16.4, D16.5, D16.6, D16.7, D16.8, D16.9, D28.0, D28.1, D28.7, D29.0, D30.2, D30.20, D30.21, D30.22, D30.4, D30.7, D30.8, D31, D31.0, D31.00, D31.01, D31.02, D31.1, D31.10, D31.11, D31.12, D31.2, D31.20, D31.21, D31.22, D31.3, D31.30, D31.31, D31.32, D31.4, D31.40, D31.41, D31.42, D31.5, D31.50, D31.51, D31.52, D31.6, D31.60, D31.61, D31.62, D31.9, D31.90, D31.91, D31.92, D32, D32.0, D32.1, D32.9, D33, D33.0, D33.1, D33.2, D33.3, D33.4, D33.7, D33.9, D35, D35.0, D35.00, D35.01, D35.02, D35.1, D35.2, D35.3, D35.4, D35.5, D35.6, D35.7, D35.8, D35.9, D36, D36.1, D36.10, D36.11, D36.12, D36.13, D36.14, D36.15, D36.16, D36.17, D36.7, D37.2, D38.2, D38.3, D38.4, D38.5, D39.2, D39.8, D41.2, D41.20, D41.21, D41.22, D41.3, D42, D42.0, D42.1, D42.9, D43, D43.0, D43.1, D43.2, D43.3, D43.4, D43.7, D43.8, D43.9, D44.1, D44.10, D44.11, D44.12, D44.2, D44.3, D44.4, D44.5, D44.6, D44.7, D44.8, D48.0, D48.1, D48.2, D48.3, D48.4, D49.6, D49.81, C49, C49.0, C49.1, C49.10, C49.11, C49.12, C49.2, C49.20, C49.21, C49.22, C49.3, C49.4, C49.5, C49.6, C49.8, C49.9`	227.1, 227.3, 227.4, 227.5, 227.6, 227.8, 227.9, 228, 228.0, 228.00, 228.01, 228.02, 228.03, 228.04, 228.09, 228.1, 228.9, 229.0, 229.8, 230.7, 230.8, 233.31, 233.32, 233.4, 233.5, 234.0, 234.5, 234.8, 235.4, 235.8, 236.1, 236.99, 237, 237.0, 237.1, 237.2, 237.3, 237.5, 237.6, 237.7, 237.70, 237.71, 237.72, 237.73, 237.79, 237.9, 238.0, 238.1, 239.2, 239.6, 171, 171.0, 171.2, 171.3, 171.4, 171.5, 171.6, 171.7, 171.8, 171.9

Abbreviations: ICD-9, International Classification of Diseases, Ninth Revision; ICD-10, International Classification of Diseases, Tenth Revision; NA, not applicable.

Supplementary Table 2: Age-standardized incidence and death rate in 2019 to 2021, annual percent change from 2000 to 2019, and annual percent change from 2000 to 2021, by region and SDI

	**Incidence** 2019 Age-standardized incidence rate (95% UI)	2020 Age-standardized incidence rate (95% UI)	2021 Age-standardized incidence rate (95% UI)	2000 to 2021 Annual Percent Change (95% CI)	*p*	2000 to 2019 Annual Percent Change (95% CI)	*p*	**Death** 2019 Age-standardized death rate (95% UI)	2020 Age-standardized death rate (95% UI)	2021 Age-standardized death rate (95% UI)	2000 to 2021 Annual Percent Change (95% CI)	*p*	2000 to 2019 Annual Percent Change (95% CI)	*p*
Both	79.12 (75.08 to 83.45)	78.59 (74.18 to 83.24)	79.91 (75.52 to 84.6)	0.4 (0.32 to 0.47)	<0.001	0.42 (0.34 to 0.5)	<0.001	25.4 (24.04 to 26.91)	25.14 (23.59 to 26.94)	25.06 (23.5 to 26.61)	-0.91 (-1.02 to -0.8)	<0.001	-0.96 (-1.03 to -0.9)	<0.001
**By Region**														
Africa	38.46 (32.67 to 44.83)	38.84 (32.45 to 45.27)	39.27 (33.36 to 45.76)	0.44 (0.33 to 0.55)	<0.001	0.38 (0.25 to 0.51)	<0.001	19.92 (16.83 to 23.34)	19.96 (16.59 to 23.34)	20.02 (16.77 to 23.64)	-0.28 (-0.35 to -0.2)	<0.001	-0.32 (-0.41 to -0.23)	<0.001
Eastern Mediterranean	52.59 (48.09 to 57.76)	52.46 (47.44 to 57.9)	53.31 (47.73 to 59.37)	1.63 (1.53 to 1.72)	<0.001	1.74 (1.65 to 1.82)	<0.001	22.56 (20.26 to 25.23)	22.44 (20.08 to 25.13)	22.55 (19.82 to 25.51)	0.4 (0.32 to 0.48)	<0.001	0.43 (0.34 to 0.52)	<0.001
Europe	118.36 (115.21 to 122.09)	112.31 (108.76 to 116.11)	112.95 (108.18 to 117.4)	-0.28 (-0.58 to 0.02)	0.067	0 (-0.16 to 0.16)	0.977	27.97 (27.39 to 28.56)	26.64 (25.87 to 27.45)	26.76 (25.6 to 27.92)	-1.7 (-1.81 to -1.58)	<0.001	-1.73 (-1.83 to -1.62)	<0.001
Region of the Americas	155.81 (146.6 to 166.72)	154.17 (144.58 to 164.8)	152.97 (143.22 to 162.91)	0.6 (0.35 to 0.84)	<0.001	0.77 (0.57 to 0.97)	<0.001	24.81 (24.18 to 25.47)	24.45 (23.55 to 25.36)	24.55 (23.41 to 25.8)	-0.64 (-0.8 to -0.48)	<0.001	-0.69 (-0.86 to -0.52)	<0.001
Southeast Asia	42.6 (39.8 to 45.75)	43.61 (40.41 to 46.92)	44.01 (40.38 to 47.82)	0.75 (0.6 to 0.9)	<0.001	0.64 (0.36 to 0.93)	<0.001	21.3 (19.87 to 22.89)	21.5 (20.04 to 23.16)	21.45 (19.7 to 23.3)	-0.24 (-0.36 to -0.13)	<0.001	-0.25 (-0.44 to -0.06)	0.01
Western Pacific	95.4 (83.91 to 107.11)	97.12 (85.71 to 111.31)	103.57 (90.99 to 118.84)	1.62 (1.38 to 1.86)	<0.001	1.41 (1.19 to 1.63)	<0.001	33.47 (29.12 to 38.35)	33.27 (28.88 to 38.45)	33.07 (28.24 to 38.5)	-0.86 (-1 to -0.73)	<0.001	-0.91 (-1.05 to -0.76)	<0.001
**By SDI**														
Low SDI	35.09 (30.15 to 39.89)	35.77 (30.4 to 40.79)	36.1 (30.96 to 41.24)	0.14 (0.01 to 0.27)	0.037	-0.01 (-0.1 to 0.08)	0.841	19.67 (16.87 to 22.4)	19.83 (16.8 to 22.89)	19.84 (16.93 to 22.78)	-0.57 (-0.66 to -0.49)	<0.001	-0.66 (-0.75 to -0.56)	<0.001
Low-middle SDI	44.07 (40.76 to 47.56)	44.79 (41.14 to 48.71)	45.21 (41.26 to 49.23)	0.92 (0.74 to 1.1)	<0.001	0.89 (0.66 to 1.12)	<0.001	22.16 (20.48 to 23.92)	22.27 (20.41 to 24.33)	22.28 (20.38 to 24.25)	0.06 (-0.11 to 0.23)	0.493	0.05 (-0.17 to 0.26)	0.675
Middle SDI	71.73 (67.25 to 77.5)	72.27 (67.06 to 77.69)	74.69 (69.27 to 80.78)	1.23 (0.99 to 1.46)	<0.001	1.18 (0.99 to 1.36)	<0.001	27.68 (25.73 to 30.01)	27.47 (25.31 to 29.76)	27.48 (25.39 to 29.93)	-0.48 (-0.66 to -0.3)	<0.001	-0.43 (-0.55 to -0.32)	<0.001
High-middle SDI	106.61 (98.49 to 116.21)	105.42 (96.53 to 115.69)	110.99 (102.38 to 121.87)	0.85 (0.71 to 0.98)	<0.001	0.82 (0.69 to 0.95)	<0.001	32.82 (30 to 35.9)	32.12 (29.01 to 35.45)	32.14 (28.99 to 35.71)	-1.3 (-1.56 to -1.04)	<0.001	-1.41 (-1.87 to -0.94)	<0.001
High SDI	175.5 (166.78 to 185.89)	172.45 (163.36 to 182.64)	171.54 (162.25 to 181.82)	0.36 (0.25 to 0.47)	<0.001	0.5 (0.27 to 0.74)	<0.001	22.45 (22.01 to 22.92)	21.74 (21.25 to 22.25)	21.4 (20.84 to 21.97)	-1.94 (-2.1 to -1.78)	<0.001	-1.87 (-1.97 to -1.77)	<0.001

Abbreviation: CI: confidence interval; SDI: sociodemographic index

Supplementary Table 3 Incidence, death, age-standardized incidence rate, age-standardized death rate, and change from 2000 to 2021 of cancer in patients aged 15-49, by 5-year age group

**Age group**	2021 ASIR (95% UI)	2000 to 2021 APC (95% CI)	***p***	2021 ASDR (95% UI)	2000 to 2021 APC (95% CI)	***p***
15-19 years	11.4 (10.39 to 12.3)	-0.13 (-0.27 to 0.01)	0.062	4.98 (4.46 to 5.43)	-1.16 (-1.23 to -1.08)	<0.001
20-24 years	18.99 (17.56 to 20.35)	0.28 (0.18 to 0.37)	<0.001	6.61 (6.03 to 7.16)	-0.84 (-0.98 to -0.7)	<0.001
25-29 years	32.9 (30.49 to 35.41)	0.53 (0.24 to 0.81)	<0.001	9.32 (8.61 to 9.98)	-0.86 (-1.15 to -0.58)	<0.001
30-34 years	58 (54.43 to 61.72)	0.5 (0.11 to 0.9)	0.012	15.68 (14.68 to 16.66)	-0.98 (-1.21 to -0.74)	<0.001
35-39 years	93.62 (87.25 to 100.4)	0.11 (-0.07 to 0.3)	0.232	26.72 (25.1 to 28.37)	-1.22 (-1.36 to -1.09)	<0.001
40-44 years	154.55 (146.05 to 164.21)	-0.06 (-0.17 to 0.05)	0.266	47.83 (44.79 to 50.84)	-1.3 (-1.52 to -1.07)	<0.001
45-49 years	238.29 (221.75 to 256.79)	-0.15 (-0.39 to 0.09)	0.232	80.34 (74.88 to 86.14)	-1.48 (-1.68 to -1.29)	<0.001

Abbreviation: APC: annual percent change; ASDR: age-standardized death rate; ASIR: age-standardized incidence rate; CI: confidence interval; UI: uncertainty interval

Supplementary Table 4: Incidence, age-standardized incidence rate, and change from 2000 to 2021 of cancer in patients aged 15-49 in females and males, by region and sociodemographic index

	**Female**			**Male**		
	2021 ASIR (95% UI)	2000 to 2021 APC (95% CI)	***p***	2021 ASIR (95% UI)	2000 to 2021 APC (95% CI)	***p***
**By Region**						
Africa	54.78 (45.56 to 63.9)	0.63 (0.51 to 0.76)	<0.001	22.85 (19.33 to 26.47)	-0.03 (-0.09 to 0.03)	0.365
Eastern Mediterranean	72.97 (63.27 to 83.03)	1.94 (1.83 to 2.05)	<0.001	35.16 (31.66 to 39.36)	1.12 (1.06 to 1.18)	<0.001
Europe	134.94 (128.54 to 140.44)	-0.15 (-0.44 to 0.14)	0.322	91.37 (87.35 to 95.63)	-0.47 (-0.87 to -0.08)	0.018
Americas	185.58 (174.12 to 196.81)	0.6 (0.37 to 0.83)	<0.001	119.83 (111.39 to 129.36)	0.6 (0.31 to 0.88)	<0.001
Southeast Asia	59.13 (53.18 to 65.77)	0.82 (0.61 to 1.02)	<0.001	29.39 (26.28 to 32.53)	0.63 (0.49 to 0.78)	<0.001
Western Pacific	114.97 (97.27 to 136.78)	1.91 (1.55 to 2.28)	<0.001	92.93 (78.68 to 110.9)	1.14 (0.96 to 1.32)	<0.001
**By SDI**						
Low SDI	50.53 (42.83 to 57.99)	0.19 (0.09 to 0.3)	<0.001	21.33 (17.94 to 24.79)	-0.07 (-0.17 to 0.02)	0.132
Low-middle SDI	61.82 (55.61 to 68.26)	1.06 (0.78 to 1.34)	<0.001	28.71 (26.08 to 31.57)	0.64 (0.54 to 0.75)	<0.001
Middle SDI	90.9 (83.26 to 100.02)	1.55 (1.33 to 1.77)	<0.001	58.95 (53.15 to 66.8)	0.91 (0.77 to 1.04)	<0.001
High-middle SDI	126.84 (114.42 to 141.92)	1.2 (0.94 to 1.45)	<0.001	96.07 (85.61 to 109.49)	0.57 (0.45 to 0.69)	<0.001
High SDI	206.59 (195.65 to 218.3)	0.41 (0.13 to 0.7)	0.004	138.64 (130.7 to 148.16)	0.3 (0.01 to 0.59)	0.04

Abbreviation: APC: annual percent change; ASIR: age-standardized incidence rate; CI: confidence interval; UI: uncertainty interval

Supplementary Table 5: Death, age-standardized death rate, and change from 2000 to 2021 of cancer in patients aged 15-49 in females and males, by region and sociodemographic index

	**Female**			**Male**		
	2021 ASDR (95% UI)	2000 to 2021 APC (95% CI)	***p***	2021 ASDR (95% UI)	2000 to 2021 APC (95% CI)	***p***
**By Region**						
Africa	24.78 (20.39 to 29.22)	-0.12 (-0.21 to -0.03)	0.008	14.98 (12.42 to 17.62)	-0.53 (-0.59 to -0.48)	<0.001
Eastern Mediterranean	26.75 (22.76 to 31.33)	0.64 (0.51 to 0.76)	<0.001	18.67 (16.48 to 21.3)	0.13 (0.03 to 0.24)	0.011
Europe	26.93 (25.42 to 28.43)	-1.4 (-1.53 to -1.27)	<0.001	26.58 (25.34 to 27.78)	-2.01 (-2.12 to -1.9)	<0.001
Americas	28.11 (26.54 to 29.86)	-0.43 (-0.6 to -0.25)	<0.001	20.93 (20.05 to 21.92)	-0.91 (-1.07 to -0.75)	<0.001
Southeast Asia	24.58 (22.05 to 27.39)	-0.22 (-0.36 to -0.09)	0.001	18.44 (16.38 to 20.44)	-0.17 (-0.35 to 0.01)	0.07
Western Pacific	26.91 (22.32 to 32.79)	-0.8 (-1.01 to -0.6)	<0.001	38.82 (31.81 to 47.66)	-0.88 (-1.21 to -0.56)	<0.001
**By SDI**						
Low SDI	24.67 (20.97 to 28.46)	-0.58 (-0.69 to -0.47)	<0.001	14.91 (12.38 to 17.58)	-0.57 (-0.66 to -0.48)	<0.001
Low-middle SDI	26.26 (23.6 to 29)	0.09 (-0.07 to 0.26)	0.267	18.33 (16.53 to 20.26)	0 (-0.14 to 0.14)	0.968
Middle SDI	26.91 (24.57 to 29.57)	-0.22 (-0.38 to -0.05)	0.009	28.04 (24.99 to 31.69)	-0.65 (-0.81 to -0.49)	<0.001
High-middle SDI	28.43 (25.28 to 32.06)	-1.11 (-1.35 to -0.87)	<0.001	35.63 (30.75 to 41.73)	-1.46 (-1.73 to -1.19)	<0.001
High SDI	22.34 (21.74 to 22.98)	-1.66 (-1.8 to -1.53)	<0.001	20.52 (19.82 to 21.3)	-2.15 (-2.3 to -2)	<0.001

Abbreviation: APC: annual percent change; ASDR: age-standardized death rate; CI: confidence interval; UI: uncertainty interval

Supplementary Table 6 Incidence, age-standardized incidence rate, and change from 2000 to 2021 of cancer in patients aged 15-49, by country

Country	2000 Incidence (95% UI)	2000 ASIR (95% UI)	2021 Incidence (95% UI)	2021 ASIR (95% UI)	2000 to 2021 APC (95% CI)	***p***
American Samoa	20 (10 to 20)	59.52 (49.99 to 69.78)	20 (20 to 20)	82.87 (65.6 to 103.94)	1.71 (1.55 to 1.88)	<0.001
Antigua and Barbuda	30 (30 to 30)	64.17 (59.83 to 69.29)	30 (30 to 40)	70.85 (65.81 to 77.22)	1.26 (0.93 to 1.59)	<0.001
Arab Republic of Egypt	11520 (10370 to 12630)	32.66 (29.41 to 35.81)	23900 (19470 to 29170)	44.72 (36.43 to 54.58)	1.21 (1.11 to 1.31)	<0.001
Argentine Republic	17100 (16550 to 17650)	94.44 (91.42 to 97.47)	21400 (20370 to 22460)	90.91 (86.54 to 95.4)	-0.24 (-0.61 to 0.13)	0.205
Australia	17130 (16190 to 18180)	177.86 (168.12 to 188.74)	18350 (17330 to 19520)	152.79 (144.29 to 162.54)	-0.69 (-0.91 to -0.48)	<0.001
Barbados	150 (140 to 160)	109.1 (102.18 to 116.72)	150 (120 to 190)	107.95 (84.96 to 137.9)	0.1 (-0.23 to 0.44)	0.542
Belize	60 (50 to 60)	48.29 (45.41 to 51.54)	130 (120 to 150)	55.61 (49.21 to 63.04)	0.9 (0.58 to 1.21)	<0.001
Bermuda	40 (30 to 40)	113.63 (100.42 to 125.4)	40 (30 to 40)	131.51 (108.44 to 161.33)	0.75 (0.62 to 0.87)	<0.001
Bolivarian Republic of Venezuela	9300 (8870 to 9730)	75.12 (71.64 to 78.58)	15400 (11830 to 19850)	117.1 (89.95 to 150.96)	2.25 (1.27 to 3.24)	<0.001
Bosnia and Herzegovina	1720 (1530 to 1930)	83.85 (74.3 to 94.06)	1210 (920 to 1470)	81.23 (61.72 to 98.68)	-0.38 (-0.8 to 0.05)	0.08
Brunei Darussalam	110 (100 to 120)	56.36 (50.95 to 61.43)	210 (180 to 240)	76.65 (66.53 to 86.71)	1.44 (0.95 to 1.94)	<0.001
Burkina Faso	1740 (1170 to 2420)	33.33 (22.54 to 46.48)	3390 (2370 to 4470)	32.87 (22.98 to 43.34)	-0.07 (-0.27 to 0.14)	0.526
Canada	23140 (22060 to 24240)	147.06 (140.21 to 154.1)	22270 (21270 to 23150)	133.85 (127.86 to 139.19)	-0.34 (-0.81 to 0.13)	0.161
Central African Republic	700 (500 to 940)	41 (29.57 to 55.5)	1070 (760 to 1470)	40.15 (28.62 to 54.96)	-0.1 (-0.25 to 0.05)	0.18
Commonwealth of Dominica	20 (20 to 20)	56.27 (50.36 to 62.02)	20 (20 to 30)	72.06 (57.3 to 88.29)	1.18 (0.9 to 1.46)	<0.001
Commonwealth of the Bahamas	170 (150 to 190)	100.16 (90.32 to 110.25)	250 (190 to 320)	119.11 (92.45 to 152.67)	0.93 (0.56 to 1.3)	<0.001
Cook Islands	10 (0 to 10)	53.24 (42.74 to 65.58)	10 (0 to 10)	73.95 (55.44 to 97.38)	1.62 (1.48 to 1.76)	<0.001
Czech Republic	7290 (6930 to 7690)	139.2 (132.33 to 146.71)	6220 (5340 to 7090)	131.87 (113.27 to 150.4)	-0.42 (-1.34 to 0.51)	0.373
Democratic People's Republic of Korea	7370 (5340 to 9740)	61.33 (44.43 to 81.12)	10660 (7770 to 14550)	77.08 (56.17 to 105.18)	1.1 (1.03 to 1.17)	<0.001
Democratic Republic of Sao Tome and Principe	20 (20 to 30)	35.25 (29.2 to 41.91)	40 (30 to 60)	38.21 (27.41 to 54.09)	0.23 (-0.31 to 0.77)	0.401
Democratic Republic of the Congo	6250 (5040 to 7800)	27.45 (22.15 to 34.29)	13600 (10060 to 17640)	31.39 (23.22 to 40.73)	0.65 (0.47 to 0.82)	<0.001
Democratic Republic of Timor-Leste	100 (80 to 120)	22.73 (18.23 to 27.66)	200 (150 to 260)	28.52 (21.21 to 37.3)	1.16 (0.28 to 2.05)	0.01
Democratic Socialist Republic of Sri Lanka	4560 (4280 to 4800)	44.06 (41.38 to 46.34)	5090 (3320 to 6990)	46.15 (30.16 to 63.38)	0.25 (-0.09 to 0.59)	0.155
Dominican Republic	1870 (1660 to 2130)	42.23 (37.53 to 48.2)	3080 (2540 to 3750)	52.54 (43.28 to 63.94)	0.98 (0.31 to 1.67)	0.004
Eastern Republic of Uruguay	1680 (1610 to 1750)	105.63 (101.33 to 109.89)	2000 (1900 to 2090)	121.74 (115.48 to 127.51)	0.62 (0.18 to 1.06)	0.006
Federal Democratic Republic of Ethiopia	14000 (12030 to 16190)	45.87 (39.43 to 53.06)	21510 (17350 to 26750)	39.09 (31.53 to 48.6)	-0.78 (-0.92 to -0.63)	<0.001
Federal Democratic Republic of Nepal	2850 (2360 to 3410)	25.04 (20.7 to 29.93)	5480 (4100 to 7170)	32.92 (24.65 to 43.04)	1.32 (1.11 to 1.52)	<0.001

Federal Republic of Germany	55770 (54040 to 57520)	138.54 (134.25 to 142.87)	41280 (39340 to 43180)	116.03 (110.58 to 121.36)	-0.86 (-1.17 to -0.55)	<0.001
Federal Republic of Nigeria	12730 (8510 to 17330)	22.15 (14.8 to 30.15)	28080 (16910 to 41570)	26.03 (15.68 to 38.53)	0.71 (0.54 to 0.89)	<0.001
Federal Republic of Somalia	2200 (1600 to 2990)	47.82 (34.71 to 64.99)	4180 (3030 to 5840)	41.94 (30.37 to 58.63)	-0.6 (-0.74 to -0.45)	<0.001
Federated States of Micronesia	30 (20 to 40)	56.03 (44.09 to 69.03)	40 (30 to 50)	65.54 (48.07 to 85.81)	0.74 (0.69 to 0.79)	<0.001
Federative Republic of Brazil	71080 (68650 to 73560)	74.61 (72.06 to 77.22)	103100 (97650 to 109070)	88.9 (84.2 to 94.05)	0.83 (0.73 to 0.93)	<0.001
French Republic	52320 (50240 to 54330)	177.35 (170.28 to 184.15)	44000 (42080 to 46340)	155.56 (148.74 to 163.8)	-0.51 (-1.19 to 0.18)	0.145
Gabonese Republic	270 (210 to 340)	45.67 (35.98 to 57.13)	450 (310 to 660)	48.91 (33.43 to 70.63)	0.27 (0.03 to 0.51)	0.029
Georgia	2580 (2260 to 2910)	107.57 (94.53 to 121.32)	1560 (1380 to 1730)	97.33 (86.55 to 108.4)	-0.3 (-1.81 to 1.24)	0.704
Grand Duchy of Luxembourg	320 (300 to 340)	143.5 (135.99 to 151.13)	320 (300 to 350)	101.73 (93.02 to 110.22)	-1.71 (-2.04 to -1.39)	<0.001
Greenland	40 (40 to 50)	138.96 (121.26 to 155.6)	30 (20 to 30)	103.95 (83.06 to 123.39)	-1.38 (-1.61 to -1.15)	<0.001
Grenada	40 (30 to 40)	69.85 (64.1 to 74.98)	50 (40 to 50)	88.14 (74.57 to 102.91)	1.15 (0.82 to 1.49)	<0.001
Guam	50 (50 to 50)	58.28 (53.72 to 62.68)	50 (50 to 60)	73.41 (65.9 to 81.65)	1.1 (-0.39 to 2.6)	0.148
Hashemite Kingdom of Jordan	1030 (830 to 1240)	41.88 (33.8 to 50.45)	3800 (3040 to 4740)	55.57 (44.38 to 69.27)	1.32 (0.83 to 1.81)	<0.001
Hellenic Republic	7600 (7290 to 7930)	134.44 (129.07 to 140.32)	6080 (5810 to 6370)	140.59 (134.26 to 147.22)	0.2 (0.05 to 0.34)	0.009
Hungary	8780 (8160 to 9350)	170.86 (158.84 to 181.99)	5590 (4830 to 6390)	128.68 (111.31 to 147.17)	-1.37 (-1.72 to -1.02)	<0.001
Independent State of Papua New Guinea	1020 (760 to 1300)	37.22 (27.51 to 47.33)	2060 (1460 to 2750)	38.5 (27.33 to 51.58)	0.14 (-0.13 to 0.41)	0.304
Independent State of Samoa	40 (30 to 50)	44.79 (31.93 to 62.86)	60 (40 to 70)	56.03 (40.66 to 74.2)	1.09 (0.97 to 1.2)	<0.001
Ireland	2770 (2640 to 2890)	135.74 (129.7 to 141.89)	3220 (3020 to 3430)	139.3 (130.99 to 148.54)	0.26 (-0.36 to 0.89)	0.412
Islamic Republic of Afghanistan	2580 (1510 to 3450)	40.24 (23.54 to 53.78)	7620 (4720 to 10600)	51.57 (31.91 to 71.73)	1.15 (0.91 to 1.38)	<0.001
Islamic Republic of Iran	13910 (13080 to 14740)	38.13 (35.86 to 40.42)	34770 (30730 to 38470)	73.36 (64.83 to 81.17)	3.11 (2.9 to 3.32)	<0.001
Islamic Republic of Mauritania	370 (260 to 480)	30.57 (21.98 to 39.52)	650 (480 to 880)	31.71 (23.26 to 42.8)	0.12 (-0.09 to 0.32)	0.272
Islamic Republic of Pakistan	31120 (27240 to 35190)	47.67 (41.72 to 53.9)	69090 (53990 to 88030)	56.63 (44.26 to 72.16)	0.81 (0.73 to 0.88)	<0.001
Jamaica	1020 (970 to 1080)	75.4 (71.22 to 79.69)	1260 (930 to 1720)	82.04 (60.47 to 112.32)	0.09 (-1.19 to 1.38)	0.894
Japan	62060 (60520 to 63580)	102.35 (99.82 to 104.86)	52680 (50990 to 54240)	103.96 (100.62 to 107.03)	0.1 (-0.17 to 0.36)	0.479
Kingdom of Bahrain	210 (190 to 230)	51.55 (46.93 to 56.95)	600 (500 to 710)	60.95 (50.9 to 72.01)	0.72 (-0.07 to 1.52)	0.076
Kingdom of Belgium	7460 (7140 to 7770)	149.47 (143.09 to 155.72)	6100 (5840 to 6440)	122.22 (116.98 to 129)	-0.95 (-1.23 to -0.67)	<0.001
Kingdom of Bhutan	90 (70 to 110)	26.62 (20.89 to 32.74)	140 (100 to 180)	32.18 (23.46 to 42.11)	0.89 (0.73 to 1.05)	<0.001
Kingdom of Cambodia	2570 (2190 to 2980)	42.58 (36.23 to 49.44)	4760 (3630 to 6480)	52.76 (40.24 to 71.82)	0.99 (0.92 to 1.06)	<0.001
Kingdom of Denmark	3880 (3760 to 4010)	151.51 (146.85 to 156.57)	2840 (2690 to 3010)	110.55 (104.53 to 117.01)	-1.48 (-1.62 to -1.35)	<0.001
Kingdom of Eswatini	310 (240 to 390)	63.57 (48.68 to 80.48)	450 (280 to 670)	73.72 (44.99 to 108.52)	0.67 (0.47 to 0.87)	<0.001
Kingdom of Lesotho	370 (280 to 470)	45.59 (33.78 to 57.52)	660 (430 to 890)	65.25 (43.05 to 88.52)	1.75 (1.25 to 2.24)	<0.001

Kingdom of Morocco	3650 (3060 to 4380)	23.5 (19.72 to 28.19)	6920 (5170 to 10260)	35.64 (26.59 to 52.84)	1.99 (1.88 to 2.11)	<0.001
Kingdom of Norway	3160 (3040 to 3300)	145.28 (139.79 to 151.68)	2900 (2780 to 3040)	116.3 (111.22 to 121.75)	-1.01 (-1.23 to -0.78)	<0.001
Kingdom of Saudi Arabia	3130 (2440 to 3850)	27.7 (21.65 to 34.07)	14750 (11220 to 19310)	58.31 (44.35 to 76.33)	3.61 (3.51 to 3.71)	<0.001
Kingdom of Spain	30020 (29070 to 30980)	140.47 (136.02 to 144.98)	25600 (24330 to 26930)	128.08 (121.72 to 134.73)	-0.36 (-0.58 to -0.15)	0.001
Kingdom of Sweden	4690 (4510 to 4860)	115.73 (111.31 to 119.95)	4030 (3670 to 4420)	89.06 (80.97 to 97.73)	-1.18 (-1.53 to -0.82)	<0.001
Kingdom of Thailand	34400 (29890 to 39450)	95.06 (82.6 to 109.02)	37690 (29310 to 47790)	118.07 (91.82 to 149.74)	1.2 (0.75 to 1.65)	<0.001
Kingdom of the Netherlands	11540 (11160 to 11950)	143.36 (138.7 to 148.49)	9230 (8760 to 9710)	124.61 (118.33 to 131.13)	-0.68 (-0.95 to -0.4)	<0.001
Kingdom of Tonga	30 (30 to 30)	64.05 (56.21 to 72.56)	40 (30 to 50)	72.56 (54.65 to 100.47)	0.59 (0.53 to 0.66)	<0.001
Kyrgyz Republic	1400 (1230 to 1580)	53.69 (47.04 to 60.73)	1960 (1630 to 2340)	56.94 (47.33 to 68.1)	0.36 (-0.06 to 0.78)	0.095
Lao People's Democratic Republic	1040 (830 to 1270)	41.44 (32.86 to 50.29)	1790 (1300 to 2350)	44.7 (32.54 to 58.6)	0.35 (0.16 to 0.55)	<0.001
Lebanese Republic	1090 (940 to 1250)	61.39 (52.88 to 70.78)	2580 (2130 to 3060)	85.59 (70.63 to 101.37)	1.6 (1.13 to 2.07)	<0.001
Malaysia	6160 (5830 to 6470)	48.09 (45.47 to 50.53)	11960 (10910 to 13200)	67.4 (61.44 to 74.37)	1.68 (1.42 to 1.95)	<0.001
Mongolia	830 (740 to 930)	62.87 (56.22 to 70.55)	1280 (1140 to 1440)	75.97 (67.33 to 85.1)	0.94 (0.37 to 1.53)	0.001
Montenegro	410 (370 to 440)	123.3 (113.34 to 133.78)	350 (310 to 410)	120.51 (104.7 to 138.73)	-0.13 (-0.87 to 0.62)	0.737
New Zealand	2980 (2820 to 3140)	152.44 (144.36 to 160.87)	3500 (3310 to 3710)	143.87 (136.21 to 152.69)	-0.27 (-0.55 to 0.01)	0.062
North Macedonia	1130 (1050 to 1210)	103.91 (96.4 to 111.99)	1050 (850 to 1270)	95.28 (77.25 to 115.52)	-0.56 (-0.94 to -0.18)	0.004
Northern Mariana Islands	30 (30 to 40)	70.83 (53.62 to 90.6)	20 (20 to 30)	94.9 (78.79 to 110.1)	0.75 (-0.1 to 1.61)	0.082
Palestine	530 (460 to 600)	38.9 (33.86 to 44.48)	1340 (1120 to 1560)	50.37 (42.29 to 58.86)	1.12 (0.66 to 1.59)	<0.001
People's Democratic Republic of Algeria	4680 (4030 to 5290)	28.05 (24.17 to 31.71)	9760 (7940 to 12090)	43.11 (35.06 to 53.41)	2.09 (2 to 2.17)	<0.001
People's Republic of Bangladesh	16550 (13720 to 19920)	25.72 (21.33 to 30.96)	31720 (24180 to 40690)	36.06 (27.49 to 46.26)	1.65 (1.35 to 1.94)	<0.001
People's Republic of China	533500 (488140 to 576190)	73.73 (67.46 to 79.63)	750580 (640900 to 886090)	113.16 (96.62 to 133.59)	2.05 (1.72 to 2.39)	<0.001
Plurinational State of Bolivia	2240 (1860 to 2690)	55.4 (46 to 66.61)	3800 (2850 to 5110)	60.8 (45.58 to 81.67)	0.46 (0.38 to 0.54)	<0.001
Portuguese Republic	7250 (6960 to 7560)	134.67 (129.28 to 140.34)	6390 (6080 to 6730)	139.43 (132.74 to 146.88)	0.12 (-0.49 to 0.72)	0.709
Principality of Andorra	60 (50 to 80)	167.82 (130.71 to 210.9)	70 (50 to 90)	158.05 (109.34 to 213.38)	-0.46 (-0.73 to -0.2)	0.001
Principality of Monaco	30 (30 to 40)	251.78 (192.37 to 325.41)	40 (30 to 50)	283.92 (202.04 to 388.27)	0.59 (0.43 to 0.75)	<0.001
Puerto Rico	1570 (1500 to 1650)	80.87 (77.11 to 85.21)	1550 (1280 to 1830)	105.71 (87.69 to 124.97)	1.72 (1.06 to 2.39)	<0.001
Republic of Albania	950 (870 to 1050)	58.24 (53.31 to 64.33)	770 (640 to 920)	61.37 (51.09 to 73.34)	0.08 (-0.27 to 0.43)	0.664
Republic of Angola	2180 (1680 to 2770)	32.48 (24.98 to 41.33)	5490 (3960 to 7410)	37.44 (27.03 to 50.53)	0.73 (0.27 to 1.18)	0.002
Republic of Armenia	1450 (1380 to 1530)	80.94 (77.24 to 85.42)	950 (820 to 1070)	65.04 (56.43 to 73.18)	-1.08 (-1.9 to -0.26)	0.01
Republic of Austria	5440 (5240 to 5650)	135.6 (130.81 to 140.92)	4350 (4130 to 4570)	107.81 (102.43 to 113.22)	-1.07 (-1.53 to -0.62)	<0.001
Republic of Azerbaijan	2550 (2310 to 2810)	56.38 (51.16 to 62.17)	2960 (2470 to 3460)	53.1 (44.4 to 62.09)	-0.15 (-0.43 to 0.13)	0.295

Republic of Belarus	5490 (5230 to 5800)	102.52 (97.52 to 108.21)	5120 (4160 to 6210)	120.36 (97.75 to 145.96)	0.23 (0.01 to 0.44)	0.04
Republic of Benin	790 (620 to 990)	27.21 (21.21 to 34.02)	1600 (1160 to 2130)	25.59 (18.52 to 34.11)	-0.29 (-0.45 to -0.13)	0.001
Republic of Botswana	350 (230 to 590)	40.2 (25.99 to 67.81)	680 (500 to 900)	49.86 (36.97 to 66.24)	0.71 (-0.19 to 1.62)	0.124
Republic of Bulgaria	4680 (4340 to 5080)	120.57 (111.9 to 130.83)	4480 (3760 to 5250)	152.58 (128.02 to 178.75)	1.29 (0.29 to 2.29)	0.011
Republic of Burundi	1360 (1060 to 1720)	48.46 (37.7 to 61.1)	2310 (1730 to 3020)	36.92 (27.66 to 48.35)	-1.29 (-1.38 to -1.2)	<0.001
Republic of C√¥te d'Ivoire	1550 (1110 to 1970)	18.56 (13.29 to 23.61)	3350 (2360 to 4550)	24.43 (17.26 to 33.22)	1.28 (0.87 to 1.69)	<0.001
Republic of Cabo Verde	80 (60 to 100)	38.08 (30.45 to 48.31)	130 (110 to 170)	42.6 (34.04 to 54.97)	0.53 (0.18 to 0.87)	0.003
Republic of Cameroon	2290 (1870 to 2760)	33.39 (27.25 to 40.17)	5310 (3630 to 7350)	34.45 (23.51 to 47.63)	0.12 (0.03 to 0.2)	0.01
Republic of Chad	880 (710 to 1100)	25.51 (20.66 to 32.05)	1940 (1430 to 2590)	26.13 (19.29 to 34.9)	0.11 (0.05 to 0.16)	0.001
Republic of Chile	5890 (5690 to 6130)	72.54 (70.06 to 75.52)	8580 (8120 to 9100)	90.24 (85.45 to 95.76)	1.15 (1.02 to 1.29)	<0.001
Republic of Colombia	14210 (13520 to 14950)	68.3 (64.97 to 71.87)	23710 (20160 to 28150)	90.81 (77.22 to 107.78)	1.77 (1.44 to 2.1)	<0.001
Republic of Costa Rica	1560 (1490 to 1640)	74.56 (71.31 to 78.16)	2870 (2560 to 3160)	114.68 (102.34 to 126.27)	1.9 (1.44 to 2.35)	<0.001
Republic of Croatia	2710 (2470 to 2970)	119.01 (108.37 to 130.6)	2150 (1850 to 2460)	117.68 (101.24 to 134.41)	-0.06 (-1.63 to 1.52)	0.936
Republic of Cuba	5810 (5560 to 6060)	93.65 (89.64 to 97.81)	5130 (4440 to 5880)	100.99 (87.31 to 115.79)	0.48 (0.27 to 0.7)	<0.001
Republic of Cyprus	400 (360 to 450)	82.85 (74.9 to 91.66)	700 (610 to 800)	99.5 (85.73 to 113.29)	0.86 (-0.16 to 1.9)	0.099
Republic of Djibouti	130 (90 to 170)	40.26 (27.88 to 53.55)	340 (230 to 500)	49.9 (32.6 to 71.68)	1.04 (0.92 to 1.15)	<0.001
Republic of Ecuador	3700 (3370 to 4030)	58.8 (53.61 to 64.03)	6160 (4800 to 7740)	65.63 (51.13 to 82.41)	0.34 (-0.52 to 1.2)	0.444
Republic of El Salvador	1860 (1720 to 1980)	66.33 (61.53 to 70.78)	2910 (2320 to 3540)	87.86 (70.12 to 107.05)	1.02 (-0.28 to 2.34)	0.126
Republic of Equatorial Guinea	100 (60 to 130)	32.91 (22.23 to 44.82)	330 (200 to 510)	40.61 (24.77 to 62.4)	1.17 (0.3 to 2.05)	0.008
Republic of Estonia	870 (820 to 920)	125.61 (118.56 to 133.19)	640 (550 to 720)	111.16 (95.57 to 125.56)	-0.56 (-0.81 to -0.32)	<0.001
Republic of Fiji	250 (230 to 270)	56.62 (51.56 to 61.33)	310 (230 to 420)	65.68 (48.62 to 88.96)	0.79 (0.33 to 1.25)	0.001
Republic of Finland	3310 (3160 to 3480)	133.8 (127.64 to 140.59)	2650 (2510 to 2800)	113.49 (107.32 to 119.72)	-0.55 (-0.87 to -0.23)	0.001
Republic of Ghana	3720 (2770 to 4550)	40.5 (30.2 to 49.53)	6450 (4840 to 8140)	36.8 (27.62 to 46.45)	-0.47 (-0.63 to -0.3)	<0.001
Republic of Guatemala	3100 (2940 to 3250)	62.65 (59.35 to 65.58)	4740 (4110 to 5420)	56.38 (48.89 to 64.44)	-0.18 (-0.41 to 0.06)	0.129
Republic of Guinea	1340 (1090 to 1600)	39.19 (31.83 to 46.92)	2460 (1750 to 3290)	40.1 (28.6 to 53.71)	0.06 (-0.03 to 0.16)	0.209
Republic of Guinea-Bissau	240 (180 to 310)	42.97 (32.41 to 54.23)	460 (340 to 600)	45.94 (33.81 to 59.54)	0.33 (0.14 to 0.52)	<0.001
Republic of Guyana	280 (240 to 320)	69.02 (58.64 to 80.15)	260 (190 to 350)	64.96 (48.69 to 87.7)	-0.28 (-0.65 to 0.09)	0.14
Republic of Haiti	2380 (1840 to 2920)	59.11 (45.85 to 72.52)	4230 (3020 to 5600)	61.65 (44.02 to 81.68)	0.22 (-0.05 to 0.48)	0.111
Republic of Honduras	1460 (1170 to 1830)	50.24 (40.31 to 62.88)	2780 (1900 to 3810)	51.43 (35.06 to 70.33)	0.09 (-0.08 to 0.27)	0.307
Republic of Iceland	180 (170 to 190)	125.07 (117.47 to 132.49)	260 (240 to 280)	157.23 (143.09 to 172.15)	1.03 (0.56 to 1.51)	<0.001
Republic of India	175750 (162740 to 188430)	33.16 (30.71 to 35.56)	310900 (279840 to 345980)	39.88 (35.89 to 44.38)	0.88 (0.68 to 1.07)	<0.001

Republic of Indonesia	49410 (42480 to 58070)	42.45 (36.5 to 49.89)	78640 (63800 to 98620)	51.29 (41.61 to 64.31)	0.94 (0.75 to 1.12)	<0.001
Republic of Iraq	4880 (3790 to 6370)	38.89 (30.2 to 50.69)	12820 (9960 to 17330)	57.85 (44.94 to 78.23)	1.91 (1.67 to 2.15)	<0.001
Republic of Italy	46370 (44820 to 47990)	167.74 (162.11 to 173.61)	37670 (35950 to 39300)	153.09 (146.1 to 159.68)	-0.52 (-1.04 to 0)	0.05
Republic of Kazakhstan	7140 (6770 to 7530)	88.54 (83.92 to 93.33)	6580 (5810 to 7270)	70.28 (62.14 to 77.67)	-1.05 (-1.17 to -0.93)	<0.001
Republic of Kenya	3740 (3030 to 4720)	25.53 (20.69 to 32.18)	9250 (7440 to 11440)	35.38 (28.45 to 43.77)	1.61 (1.28 to 1.95)	<0.001
Republic of Kiribati	30 (20 to 40)	68.26 (55.09 to 83.82)	40 (30 to 60)	70.43 (51.65 to 95.51)	0.12 (0.04 to 0.21)	0.004
Republic of Korea	21480 (20200 to 22590)	78.42 (73.75 to 82.49)	23030 (21290 to 25080)	94.84 (87.65 to 103.28)	0.95 (0.75 to 1.14)	<0.001
Republic of Latvia	1100 (1050 to 1160)	93.87 (89.34 to 99.15)	820 (710 to 920)	103.99 (90.68 to 116.67)	0.23 (0 to 0.46)	0.05
Republic of Liberia	350 (250 to 470)	26.58 (18.91 to 35.31)	1020 (670 to 1410)	36.25 (24.03 to 50.17)	1.56 (1.16 to 1.96)	<0.001
Republic of Lithuania	1930 (1830 to 2010)	108.97 (103.84 to 114.01)	1360 (1180 to 1510)	117.42 (102.4 to 130.47)	0.17 (-0.72 to 1.08)	0.706
Republic of Madagascar	2980 (2530 to 3490)	40.87 (34.61 to 47.75)	6130 (4510 to 8340)	43.4 (31.94 to 59.06)	0.28 (0.02 to 0.53)	0.032
Republic of Malawi	2440 (1940 to 2990)	48.24 (38.28 to 59.18)	5130 (3800 to 6750)	52.97 (39.27 to 69.76)	0.54 (0.05 to 1.02)	0.031
Republic of Maldives	40 (30 to 40)	25.93 (21.87 to 30.19)	80 (70 to 100)	24.61 (19.51 to 29.3)	-0.39 (-0.62 to -0.17)	0.001
Republic of Mali	1790 (1440 to 2110)	37.83 (30.4 to 44.56)	3700 (2840 to 4730)	35.21 (27.07 to 45.02)	-0.37 (-0.47 to -0.27)	<0.001
Republic of Malta	230 (210 to 240)	112.49 (106.58 to 119.38)	260 (230 to 280)	132.63 (120.47 to 145.71)	0.77 (-0.01 to 1.55)	0.053
Republic of Mauritius	390 (370 to 400)	55.49 (52.63 to 58.28)	500 (450 to 530)	78.02 (69.76 to 82.89)	1.6 (0.47 to 2.74)	0.005
Republic of Moldova	1830 (1730 to 1920)	80.67 (76.26 to 84.97)	1470 (1320 to 1650)	82.4 (73.51 to 92.28)	-0.04 (-0.27 to 0.19)	0.748
Republic of Mozambique	2990 (2190 to 4240)	37.82 (27.72 to 53.62)	6430 (4370 to 8880)	45.06 (30.61 to 62.27)	0.88 (0.65 to 1.11)	<0.001
Republic of Namibia	380 (310 to 460)	42.66 (34.12 to 51.5)	710 (480 to 1020)	55.38 (37.48 to 78.78)	1.44 (1.07 to 1.82)	<0.001
Republic of Nauru	0 (0 to 10)	81.33 (58.77 to 103)	0 (0 to 10)	83.09 (58.67 to 110.27)	0.08 (0.03 to 0.14)	0.005
Republic of Nicaragua	1170 (1080 to 1260)	48.21 (44.56 to 52.2)	1960 (1680 to 2330)	54.64 (46.87 to 64.89)	0.46 (-0.64 to 1.57)	0.416
Republic of Niue	0 (0 to 0)	62.62 (47.26 to 80.26)	0 (0 to 0)	78.25 (62.61 to 100.17)	1.24 (0.76 to 1.73)	<0.001
Republic of Palau	10 (10 to 20)	103.14 (81.68 to 128.5)	10 (10 to 10)	114.97 (91.01 to 142.13)	0.54 (0.39 to 0.68)	<0.001
Republic of Panama	1000 (940 to 1050)	65.41 (61.88 to 68.82)	1970 (1630 to 2360)	91.13 (75.22 to 109.04)	1.62 (1.48 to 1.77)	<0.001
Republic of Paraguay	1250 (1060 to 1480)	49.47 (42.03 to 58.54)	2180 (1660 to 2920)	56.37 (42.9 to 75.76)	0.79 (0.5 to 1.08)	<0.001
Republic of Peru	7180 (6200 to 8310)	54.6 (47.15 to 63.17)	14170 (10880 to 17630)	73.33 (56.27 to 91.22)	1.3 (0.42 to 2.18)	0.004
Republic of Poland	22090 (21520 to 22670)	108.23 (105.44 to 111.11)	17460 (15930 to 18960)	97.44 (88.9 to 105.77)	-0.4 (-0.72 to -0.08)	0.013
Republic of Rwanda	2260 (1930 to 2640)	60.08 (51.41 to 70.32)	3200 (2420 to 4210)	46.65 (35.29 to 61.42)	-1.19 (-1.35 to -1.02)	<0.001
Republic of San Marino	20 (20 to 20)	154.54 (135.23 to 174.28)	20 (10 to 20)	124.96 (79.02 to 177.62)	-1.28 (-1.64 to -0.92)	<0.001
Republic of Senegal	1140 (860 to 1490)	24.78 (18.75 to 32.4)	2410 (1840 to 3180)	31.18 (23.78 to 41.15)	0.53 (0.28 to 0.78)	<0.001
Republic of Serbia	5930 (5510 to 6380)	121.2 (112.6 to 130.38)	4440 (3730 to 5120)	104.78 (88 to 120.69)	-0.74 (-1.36 to -0.11)	0.021

Republic of Seychelles	40 (30 to 40)	79.2 (73.21 to 85.73)	50 (40 to 60)	91.12 (80.99 to 102.69)	1.12 (0.89 to 1.35)	<0.001
Republic of Sierra Leone	480 (310 to 680)	24.55 (15.59 to 34.41)	1260 (870 to 1700)	28.4 (19.57 to 38.32)	0.64 (0.3 to 0.98)	<0.001
Republic of Singapore	1680 (1620 to 1740)	65.76 (63.42 to 68.39)	2050 (1940 to 2160)	68.58 (64.89 to 72.55)	0.32 (-0.26 to 0.89)	0.279
Republic of Slovenia	1540 (1460 to 1630)	145.18 (137.67 to 153.7)	1180 (990 to 1370)	133.96 (112.95 to 156.5)	-0.54 (-2.33 to 1.28)	0.558
Republic of South Africa	14120 (13230 to 15070)	57.75 (54.13 to 61.67)	24140 (21680 to 26540)	77.49 (69.6 to 85.19)	1.52 (1.09 to 1.95)	<0.001
Republic of South Sudan	1040 (740 to 1450)	30.81 (21.86 to 42.91)	2100 (1520 to 2840)	47.26 (34.25 to 63.91)	2.11 (1.54 to 2.68)	<0.001
Republic of Sudan	3300 (2370 to 4380)	26.73 (19.23 to 35.55)	7760 (5080 to 11090)	34.71 (22.73 to 49.61)	1.26 (1.15 to 1.36)	<0.001
Republic of Suriname	130 (120 to 150)	54.58 (48.62 to 60.36)	200 (160 to 240)	68.91 (55.91 to 83.88)	1.15 (0.41 to 1.89)	0.002
Republic of Tajikistan	1240 (1110 to 1390)	40.5 (36.29 to 45.45)	1970 (1670 to 2320)	38.29 (32.51 to 45.11)	-0.26 (-0.36 to -0.17)	<0.001
Republic of the Congo	750 (570 to 980)	47.36 (36.22 to 61.93)	1620 (1130 to 2330)	57.33 (40.26 to 82.77)	0.99 (0.75 to 1.23)	<0.001
Republic of the Gambia	170 (130 to 220)	27.04 (20.49 to 34.21)	400 (290 to 540)	33.54 (24.24 to 45.29)	1.39 (0.69 to 2.1)	<0.001
Republic of the Marshall Islands	10 (10 to 20)	53.93 (41.35 to 65.57)	20 (20 to 30)	69.22 (49.61 to 90.25)	1.2 (1.16 to 1.24)	<0.001
Republic of the Niger	1080 (820 to 1440)	22.41 (17.05 to 29.77)	2040 (1430 to 2830)	19.65 (13.77 to 27.31)	-0.67 (-0.95 to -0.38)	<0.001
Republic of the Philippines	19340 (17440 to 20970)	47.98 (43.27 to 52.02)	33890 (28230 to 40270)	56.45 (47.02 to 67.07)	0.71 (0.44 to 0.98)	<0.001
Republic of the Union of Myanmar	12910 (10020 to 16100)	52 (40.37 to 64.88)	14600 (11010 to 19020)	49.68 (37.46 to 64.71)	-0.22 (-0.29 to -0.15)	<0.001
Republic of Trinidad and Tobago	500 (470 to 530)	68.87 (65.31 to 72.64)	670 (490 to 880)	97.47 (71.72 to 127.48)	2.21 (1.27 to 3.17)	<0.001
Republic of Tunisia	2240 (1870 to 2690)	41.68 (34.8 to 50.03)	3920 (2920 to 5080)	64.91 (48.26 to 84.05)	2.15 (2.07 to 2.22)	<0.001
Republic of Turkey	21290 (18770 to 23840)	58.35 (51.44 to 65.31)	40090 (33040 to 48060)	91.25 (75.21 to 109.4)	2.1 (1.9 to 2.3)	<0.001
Republic of Uganda	5360 (4280 to 6450)	51.9 (41.38 to 62.37)	10730 (7650 to 14000)	53.39 (38.06 to 69.62)	0.04 (-0.09 to 0.16)	0.577
Republic of Uzbekistan	5920 (5460 to 6440)	45.19 (41.71 to 49.16)	9070 (7660 to 10390)	50.87 (42.94 to 58.28)	0.55 (0.23 to 0.87)	0.001
Republic of Vanuatu	40 (30 to 50)	40.52 (29.79 to 53.37)	70 (50 to 90)	45.76 (34.92 to 57.71)	0.5 (0.26 to 0.74)	<0.001
Republic of Yemen	1700 (1230 to 2290)	20.84 (15.03 to 27.98)	4510 (2990 to 6450)	26.98 (17.88 to 38.56)	1.37 (0.7 to 2.04)	<0.001
Republic of Zambia	2330 (1900 to 2820)	52.34 (42.68 to 63.25)	6360 (3980 to 10430)	65.87 (41.24 to 108)	1.12 (0.86 to 1.39)	<0.001
Republic of Zimbabwe	3470 (2440 to 4490)	59.51 (41.79 to 76.94)	6260 (4470 to 8500)	80.84 (57.72 to 109.75)	1.49 (0.98 to 2.01)	<0.001
Romania	12410 (11770 to 13100)	106.92 (101.4 to 112.86)	9650 (8470 to 10930)	115.89 (101.65 to 131.25)	0.13 (-0.39 to 0.65)	0.616
Russian Federation	85610 (83860 to 87140)	106.08 (103.92 to 107.99)	84220 (76640 to 91070)	124.88 (113.64 to 135.05)	0.94 (0.59 to 1.3)	<0.001
Saint Kitts and Nevis	20 (10 to 20)	63.74 (55.43 to 72.65)	20 (20 to 20)	61.15 (48.03 to 78.59)	-0.32 (-0.55 to -0.08)	0.008
Saint Lucia	60 (60 to 60)	73.19 (68.65 to 78.47)	90 (70 to 110)	94.19 (77.87 to 116.05)	1.02 (0.52 to 1.52)	<0.001
Saint Vincent and the Grenadines	50 (50 to 50)	84.23 (78.45 to 90.39)	60 (50 to 70)	108.95 (94.12 to 126.98)	1.05 (0.18 to 1.93)	0.018
Slovak Republic	3390 (3210 to 3550)	115.99 (110.11 to 121.48)	3090 (2650 to 3570)	119.64 (102.34 to 138.15)	0.01 (-0.85 to 0.87)	0.984
Socialist Republic of Viet Nam	16940 (13520 to 20750)	38.84 (30.98 to 47.56)	32590 (25040 to 42880)	62.57 (48.08 to 82.33)	2.29 (2.24 to 2.35)	<0.001

Solomon Islands	90 (60 to 120)	42.01 (29.04 to 56.62)	200 (150 to 270)	58.27 (42.62 to 79.3)	1.64 (1.41 to 1.86)	<0.001
State of Eritrea	940 (670 to 1230)	49.93 (35.7 to 65.38)	1890 (1310 to 2600)	55.16 (38.24 to 75.81)	0.49 (0.37 to 0.6)	<0.001
State of Israel	3290 (3150 to 3450)	103.46 (99.04 to 108.38)	3970 (3740 to 4200)	88.28 (83.21 to 93.44)	-0.83 (-1.32 to -0.34)	0.001
State of Kuwait	660 (620 to 700)	54.86 (51.85 to 57.94)	1930 (1660 to 2250)	63.49 (54.54 to 73.85)	0.79 (0.4 to 1.18)	<0.001
State of Libya	1300 (1160 to 1480)	46.88 (42.07 to 53.62)	3470 (2640 to 4570)	84.13 (64.06 to 110.8)	2.83 (2.45 to 3.2)	<0.001
State of Qatar	160 (140 to 200)	43.21 (35.81 to 51.34)	1040 (800 to 1310)	47.29 (36.58 to 59.91)	0.27 (-0.38 to 0.91)	0.422
Sultanate of Oman	270 (220 to 320)	21.32 (17.44 to 25.78)	720 (590 to 880)	24.09 (19.58 to 29.51)	0.26 (-0.19 to 0.7)	0.257
Swiss Confederation	5730 (5540 to 5920)	157.55 (152.4 to 162.8)	4060 (3820 to 4330)	101.4 (95.34 to 108.09)	-2.22 (-2.7 to -1.74)	<0.001
Syrian Arab Republic	2650 (2270 to 3050)	32.14 (27.46 to 36.94)	3650 (2730 to 4980)	51.37 (38.5 to 70.17)	2.32 (1.62 to 3.03)	<0.001
Taiwan (Province of China)	15420 (14960 to 15890)	119.03 (115.5 to 122.65)	15970 (14850 to 16850)	140.45 (130.56 to 148.17)	0.82 (0.6 to 1.04)	<0.001
Togolese Republic	640 (490 to 800)	27.78 (21.43 to 35.17)	1470 (1010 to 1960)	35.25 (24.31 to 47.17)	1.17 (0.9 to 1.45)	<0.001
Tokelau	0 (0 to 0)	57.13 (39.79 to 76.36)	0 (0 to 0)	78.1 (65.19 to 98.08)	1.8 (1.22 to 2.38)	<0.001
Turkmenistan	1030 (960 to 1120)	47.05 (43.81 to 50.84)	1750 (1360 to 2240)	65.45 (50.81 to 83.85)	1.61 (1.04 to 2.19)	<0.001
Tuvalu	0 (0 to 0)	66.21 (53.25 to 81.44)	0 (0 to 0)	56.97 (44.3 to 72.35)	-0.72 (-0.79 to -0.64)	<0.001
Ukraine	28780 (27590 to 29830)	113.12 (108.44 to 117.24)	19300 (13500 to 25070)	95.4 (66.74 to 123.95)	-1.14 (-1.93 to -0.35)	0.005
Union of the Comoros	120 (90 to 150)	46.98 (33.11 to 59.11)	220 (170 to 280)	55.08 (42.2 to 71.02)	0.99 (-0.13 to 2.12)	0.085
United Arab Emirates	1010 (850 to 1210)	43.7 (36.58 to 52.19)	3570 (2850 to 4360)	52 (41.49 to 63.51)	0.96 (-0.03 to 1.96)	0.058
United Kingdom of Great Britain and Northern Ireland	39380 (38040 to 40720)	139.05 (134.32 to 143.81)	39370 (37990 to 40730)	129.75 (125.21 to 134.22)	-0.4 (-1 to 0.19)	0.183
United Mexican States	32080 (30770 to 33350)	60.54 (58.07 to 62.94)	56340 (50060 to 63350)	82.29 (73.12 to 92.52)	1.49 (1.31 to 1.66)	<0.001
United Republic of Tanzania	6460 (5060 to 8240)	41.2 (32.27 to 52.5)	12560 (9270 to 16190)	44.55 (32.87 to 57.42)	0.37 (0.28 to 0.46)	<0.001
United States of America	373350 (341040 to 411510)	258.82 (236.42 to 285.27)	472560 (432770 to 516740)	310.89 (284.72 to 339.96)	0.92 (0.54 to 1.3)	<0.001
United States Virgin Islands	60 (50 to 60)	105.77 (90.44 to 122.4)	30 (30 to 40)	100.21 (78.81 to 127.82)	-0.21 (-0.89 to 0.47)	0.542

Abbreviation: APC: annual percent change; ASIR: age-standardized incidence rate; CI: confidence interval; UI: uncertainty interval
